# Dimeric and high-resolution structures of Chlamydomonas Photosystem I from a temperature-sensitive Photosystem II mutant

**DOI:** 10.1038/s42003-021-02911-7

**Published:** 2021-12-09

**Authors:** Ido Caspy, Tom Schwartz, Vinzenz Bayro-Kaiser, Mariia Fadeeva, Amit Kessel, Nir Ben-Tal, Nathan Nelson

**Affiliations:** Department of Biochemistry and Molecular Biology, The George S. Wise Faculty of Life Sciences, Tel Aviv University, Tel Aviv, 69978 Israel

**Keywords:** Cryoelectron microscopy, Photosystem I

## Abstract

Water molecules play a pivotal functional role in photosynthesis, primarily as the substrate for Photosystem II (PSII). However, their importance and contribution to Photosystem I (PSI) activity remains obscure. Using a high-resolution cryogenic electron microscopy (cryo-EM) PSI structure from a *Chlamydomonas reinhardtii* temperature-sensitive photoautotrophic PSII mutant (TSP4), a conserved network of water molecules - dating back to cyanobacteria - was uncovered, mainly in the vicinity of the electron transport chain (ETC). The high-resolution structure illustrated that the water molecules served as a ligand in every chlorophyll that was missing a fifth magnesium coordination in the PSI core and in the light-harvesting complexes (LHC). The asymmetric distribution of the water molecules near the ETC branches modulated their electrostatic landscape, distinctly in the space between the quinones and FX. The data also disclosed the first observation of eukaryotic PSI oligomerisation through a low-resolution PSI dimer that was comprised of PSI-10LHC and PSI-8LHC.

## Abstract (web summary)

Caspy et al. report the structure of PSI from a temperature-sensitive photoautotrophic PSII mutant of Chlamydomonas reinhardtii (TSP4), and report the distribution of conserved water molecules in the structure from cyanobacterial to higher plant PSI. They suggest that the asymmetric distribution of water molecules near the electron transfer chain modulates the electron transfer from quinones to FX.

## Introduction

Biological energy conversion relies on a sequence of redox reactions within the membrane-embedded electron transport chains (ETC) of chloroplasts and mitochondria. In plants, the photosynthetic electron transport between the two photosystems – Photosystem I and II - must cover distances of a few hundred nanometres. This is due to the unique architectural features of the photosynthetic thylakoid membranes, which fold into stacked grana and unstacked stroma thylakoids^1^. However, the thylakoid architecture and the configuration of the protein complexes are subjected to constant modulation in response to environmental and physiological stresses^2–4^. Plastoquinone and plastocyanin shuttle electrons from Photosystem II (PSII) to Photosystem I (PSI), one within the hydrophobic milieu of the membrane, and the second in the hydrophilic environment of the lumen^5^. Plastocyanin exploits specific binding sites on its electron donor – cytochrome f - and its electron acceptor – PSI^6^.

The unicellular green alga *Chlamydomonas reinhardtii* has long been used as a model system to study photosynthesis and chloroplast biogenesis, as well as for the assembly of the membrane complexes^7^. It has frequently been referred to as the “yeast of photosynthetic organisms”. Similar to yeast, the development of tools, such as transformations and the generation of site-specific mutations, has opened up a variety of research avenues. In yeast, temperature-sensitive mutants have been widely used to study structure, biogenesis, and the functions of a large variety of essential proteins^8^. The approach of using negative selection, where the desired mutant does not grow under the restricting condition, is rarely used. Yet, this tedious “negative selection” has yielded mutants that would have been otherwise neglected^9^. Despite its advantages, this method has seldom been exploited in the study of photosynthesis. Notwithstanding the last remark, negative selection has been used to isolate temperature-sensitive-photoautotrophic (TSP) mutants in *C. reinhardtii*^10,11^. Roughly, 12,000 mutagenised colonies that were grown from UV-exposed cells were screened, and unexpectedly, many TS mutants were identified. The thorough phenotyping of all of the TSP mutants enabled the selection of four of the most interesting mutants, according to a variety of criteria. The selected mutants were called TSP1, TSP2, TSP3 and TSP4, and the latter mutant TSP4 was used in this study^10^. TSP4 has been demonstrated to completely degrade PSII upon a high-temperature treatment. The identified mutation on the PsbO gene caused a single amino acid change Pro101His in PsbO, which resulted in a temperature-sensitive PSII^12^. The elevated temperature resulted in the release of the mutated PsbO from the PSII complex and caused the entire complex to destabilise and degrade. In this work, the lack of PSII in the TSP4 cells that were grown at 37 °C was exploited to evaluate how PSII absence attuned to the structure and function of PSI. TSP4 PSI was isolated, purified and its structures were determined at 2.54 Å and 3.15 Å resolution using cryogenic electron microscopy (cryo-EM). Additionally, a low-resolution reconstruction of a dimeric PSI was identified in the preparation, the first report of PSI oligomerization in eukaryotes. The high resolution of TSP4 PSI revealed over 330 water molecules within the complex, many of them conserved across cyanobacteria and eukaryotes. The contribution and effect of these water molecules on the electrostatic landscape of PSI reaction centre were assessed, implying that they act as electrostatic modulators.

## Results and discussion

**Fig. 1:**
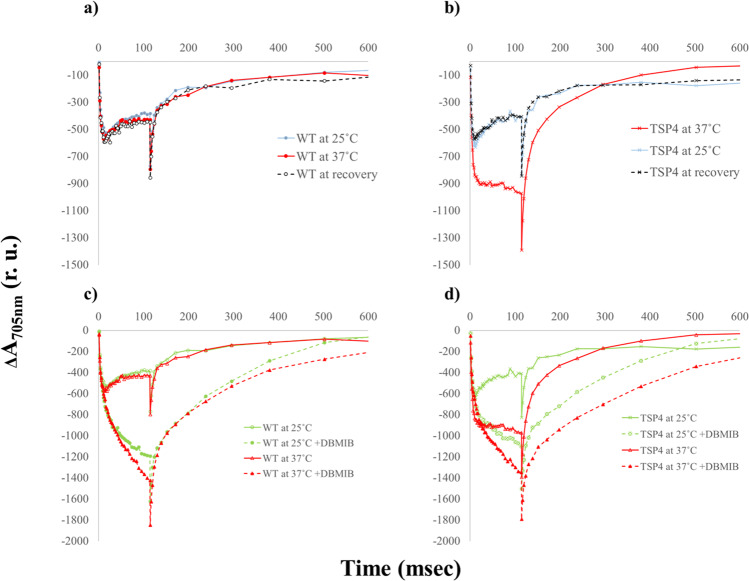
Light-induced P700 photo-oxidation and P700 + reduction of the WT and TSP4 C. reinhardtii cells. P700 kinetics of the wild-type cells (**a**) and the TSP4 cells (**b**) that were grown overnight at 25 °C (blue), or at non-permissive temperatures of 37 °C (red), which were subsequently transferred back to recover at 25 °C (grey dashed line). P700 kinetics of the wild-type cells (**c**) and the TSP4 cells (**d**) that were grown overnight at 25 °C (green), or at non-permissive temperatures of 37 °C (orange), in the absence or presence of DBMIB (dashed line).

The oxygenic photosynthetic chain can function under two different modes: the linear electron flow, which produces reducing power and ATP, and the cyclic electron flow, which only produces ATP. In both the linear and cyclic electron transport, the sole electron source for PSI is the b6f complex, which is mediated by plastocyanin or cytochrome c6. Plastocyanin is a luminal mobile electron carrier that shuttles electrons from b6f to PSI, and by restricting plastocyanin’s mobility P700^+^ reduction is hampered^6,13^. Under ambient conditions and moderate light intensities, P700 remains in its reduced form, due to the faster electron transport from the b6f complex when compared to the rate of the light-induced P700 oxidation. This occurrence is dependent on the electrons supplied by PSII, or on reduced ferredoxin. This phenomenon was clearly demonstrated by monitoring the light-induced P700 oxidation in *C. reinhardtii* cells^14^. The fact that the b6f inhibitor dibromothymoquinone (DBMIB) blocked PSI reduction more efficiently than DCMU, suggests a significant supply of electrons from sources different than PSII, such as the reduced ferredoxin or NADPH^15^. This simple and straightforward approach was incorporated to track the light-induced P700 oxidation in the *C. reinhardtii* wild type (WT) and TSP4 mutant cells. P700 oxidation and reduction kinetics have been studied extensively in various *C. reinhardtii* strains^13,16^. These works have determined P700 kinetics both during linear electron transfer (LEF) and during cyclic electron transfer (CEF). The latter is induced when DCMU prevents electron transfer from PSII to cytochrome b6f, but keeps both photosystems functional. LEF and CEF occur simultaneously in the thylakoid membrane and are used to regulate the ATP:NAPDH ratio generated during photosynthesis^16^. The wild type cells exhibited similar kinetic properties, regardless of the growth temperature (Fig. 1a). The TSP4 cells that were grown at 25 °C showed similar kinetic properties when compared to the WT cells (Fig. 1b). However, when adapted overnight at 37 °C to eliminate PSII, the TSP4 cells showed a much larger P700 photo oxidation signal, due to the lack of electron supply from PSII when illuminated. The light-induced P700 signal of TSP4 when it was adapted to 37 °C was similar - but not identical - to those of the wild-type cells in the presence of DBMIB (Fig.1c). The difference in the dark re-reduction kinetics caused by DBMIB in the TSP4 cells that were adapted to 37 °C implied an electron supply by components involved in the cyclic electron flow (Fig. 1d). No changes in PSI content or activity were detected under the above conditions. The stationary stage observed during the illumination is likely caused by a population of plastocyanin that is pre-bound to PSI. Once the light is turned on, these plastocyanin molecules reduce P700^+^. After this initial plastocyanin population is dissociated, we observed the sharp decrease, as the release of plastocyanin from PSI is nearly six times slower than its binding to PSI^17^. DBMIB prevented electron transfer from cytochrome b6f to plastocyanin, causing P700^+^ reduction to occur slower and so the stationary phase cannot occur (Fig. 1c, d). The observed differences in P700 oxidation when DBMIB was added to TSP4 cells adapted to 37 °C compared to 25 °C suggested a change, either in the composition of PSI or its interaction with other proteins, since electrons were still delivered to PSI even though PSII had degraded and plastocyanin was unable to receive electrons from cytochrome b6f. Thus, the authors set out to solve the structure of TSP4 PSI by cryo-EM.

**Fig. 2:**
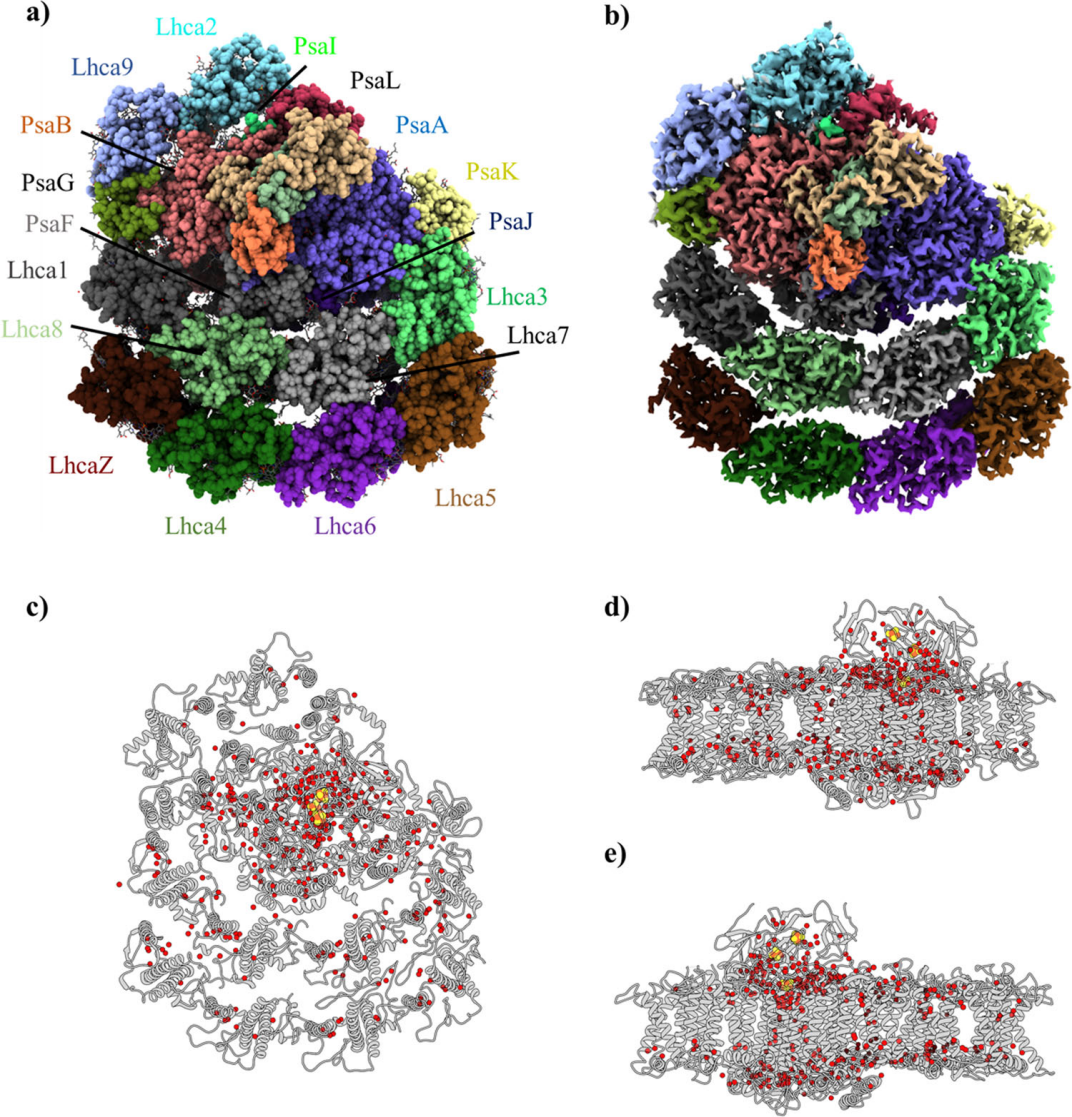
Structure of C. reinhardtii TSP4–10LHC PSI at 2.54 Å and the distribution of water molecules within the complex. **a** TSP4–10LHC 2.54 Å PSI model. The subunits are annotated according to their corresponding colours. The stromal subunits (PsaC, PsaD and PsaE) are shown but are not annotated for clarity. **b** Cryo-EM 2.54 Å map of TSP4–10LHC, coloured according to the subunits in panel **a**. Stromal (**c**) and the membrane plane view (**d**) – from the PsaK pole; **e** – from the PsaG pole of the TSP4–10LHC water molecules. The water molecules are shown as red spheres, the iron-sulphur clusters as red-brown spheres, and PSI displayed as grey cartoons.

The cryo-EM structures at a moderated resolution (2.90–3.30 Å) of *Chlamydomonas* PSI have been published previously^18,19^. The current authors decided to exploit the absence of PSII in the TSP4 *C. reinhardtii* cells that were grown overnight at a non-permissive temperature, to obtain two PSI structures at high resolutions, for the 10 and 8 light-harvesting complexes (LHCs), respectively. PSI was isolated and purified, as described in the Methods Section, and the structures of the isolated PSI were solved by cryo-EM to 2.54 Å and 3.15 Å resolution (TSP4–10LHC and TSP4–8LHC, respectively; Supplementary Figs. 1, 2 and Table 1). The structure and cryo-EM map of PSI from the TSP4 *C. reinhardtii* mutant that was grown at an elevated temperature is presented (Fig. 2a, b). For the most part, it was quite similar to the one that was obtained from the wild-type cells^18,19^, albeit with a much-improved resolution and map-density clarity (Supplementary Figs. 3 and 4). This was evident by the identification of 337 water molecules, which were detected mostly in the membrane region of the complex (Fig. 2c–e). The structure demonstrated that a water molecule coordinated with practically every chlorophyll molecule in which magnesium (Mg) was not coordinated by an amino acid. Moreover, in some chlorophylls, two water molecules were present forming an L shape of Mg-H_2_O-H_2_O, previously identified in high resolution PSI and PSII structures^20,21^, which filled up the space that was not occupied by the neighbouring amino acids (Supplementary Fig. 5 and Supplementary Table 1). Theoretical calculations propose that the Mg-H_2_O coordination can alter the absorption profile of chlorphyll a molecules, and might be further affected by the number of water molecules associated with the chlorophylls^22^. Out of 243 chlorophylls identified in TSP4–10LHC, 9% were coordinated by a single H_2_O molecule, 13% were coordinated by the L-shaped H_2_O-H_2_O (Supplementary Table 1), 5% were coordinated by phosphatidylglycerols and 73% by an amino acid. Chlorophyll axial ligands were proposed to induce a red-shift in their absorption spectra^23^.

**Table 1:** Cryo-EM data collection, refinement and validation statistics.

	TSP4–10LHC (EMDB-12180) (PDB 7BGI)	TSP4–8LHC (EMDB-12227) (PDB 7BLX)
Data collection and processing		
Magnification	165,000	165,000
Voltage (kV)	300	300
Electron exposure (e–/Å^2^)	46.80	46.80
Defocus range (μm)	0.9–3.0	0.9–3.0
Pixel size (Å)	0.827	0.827
Symmetry imposed	C1	C1
Initial particle images (no.)	1,208,518	1,208,518
Final particle images (no.)	101,572	17,311
Map resolution (Å)	2.54	3.15
FSC threshold	0.143	0.143
Map resolution range (Å)	2.3–4.5	2.8–5.0
Refinement		
Initial model used (PDB code)	6JO5	6JO5
Model resolution (Å)	2.90	2.90
FSC threshold	0.143	0.143
Model resolution range (Å)	2.5–4.5	2.5–4.5
Map sharpening *B* factor (Å^2^)	−61.60	−63.39
Model composition		
Non-hydrogen atoms	52213	47524
Protein residues	21	19
Ligands	354	317
* B* factors (Å^2^)		
Protein	9.87–61.77	4.14–54.08
Ligand	13.38–74.87	6.91–63.26
R.m.s. deviations		
Bond lengths (Å)	0.006	0.007
Bond angles (°)	1.981	1.976
Validation		
MolProbity score	1.64	1.67
Clashscore	8.36	8.78
Poor rotamers (%)	0.00	0.00
Ramachandran plot		
Favoured (%)	96.85	96.81
Allowed (%)	3.05	3.17
Disallowed (%)	0.09	0.03

The much-improved density of the map revealed several structural features of *C. reinhardtii* PSI that were noteworthy. The position and orientation of all of the 242 chlorophyll molecules, including the 25 chlorophyll b molecules, which were found in the light-harvesting complexes, were built into the model. TSP4 PSI contained less chlorophyll b molecules in the first LHC belt, compared to the pea crystal structure and *D. salina* PSI^24,25^ (6 chlorophyll b molecules in TSP4, 13 in pea and 10 in *D. salina*). The increased ratio of chlorophyll a in the LHC belt may improve the efficiency of excitation energy transfer from the first LHC belt to PSI core in TSP4. Several phytol chains were extended and the positions of some of them were reassigned. The comparison with the new PSI-LHCI-LHCII^26^ revealed a substantial rotation in Lhca2 helices I, II and III, which was caused by the PsaH insertion, moving by 8, 10 and 7 Å, respectively. Transmembrane helix IV remained in its location and it may serve as a pivotal axis for Lhca2 movement. Lhca9 drifted away from PsaB/PsaG to a lesser extent, namely, 3–5 Å (Supplementary Fig. 6). In agreement with^19^ and contrary to^18^, no trace of PsaH was detected in the present structure. Consequently, Lhca2 was markedly moved towards Lhca9 and PsaB (Supplementary Fig. 6). Since PsaH was sandwiched between PsaL and PsaB, in all of the solved structures of PSI from the different organisms, its absence was likely to present a significant functional property. It was proposed that one of the driving forces for the PsaH evolution was its ability to prevent PSI oligomerisation^27^. This was evident by the absence of PsaH in cyanobacterial PSI^21,28^, where the trimerisation of PSI seems to be rely mainly on interactions of PsaL from adjacent monomers. PsaH, composed of one transmembrane helix and one membrane parallel helix, restricts the association of neighbouring PsaL subunits. The role of PsaL in eukaryotes has altered slightly, and together with PsaH and PsaO regulates the association of LHCII to PSI during state transition^26^.

**Fig. 3:**
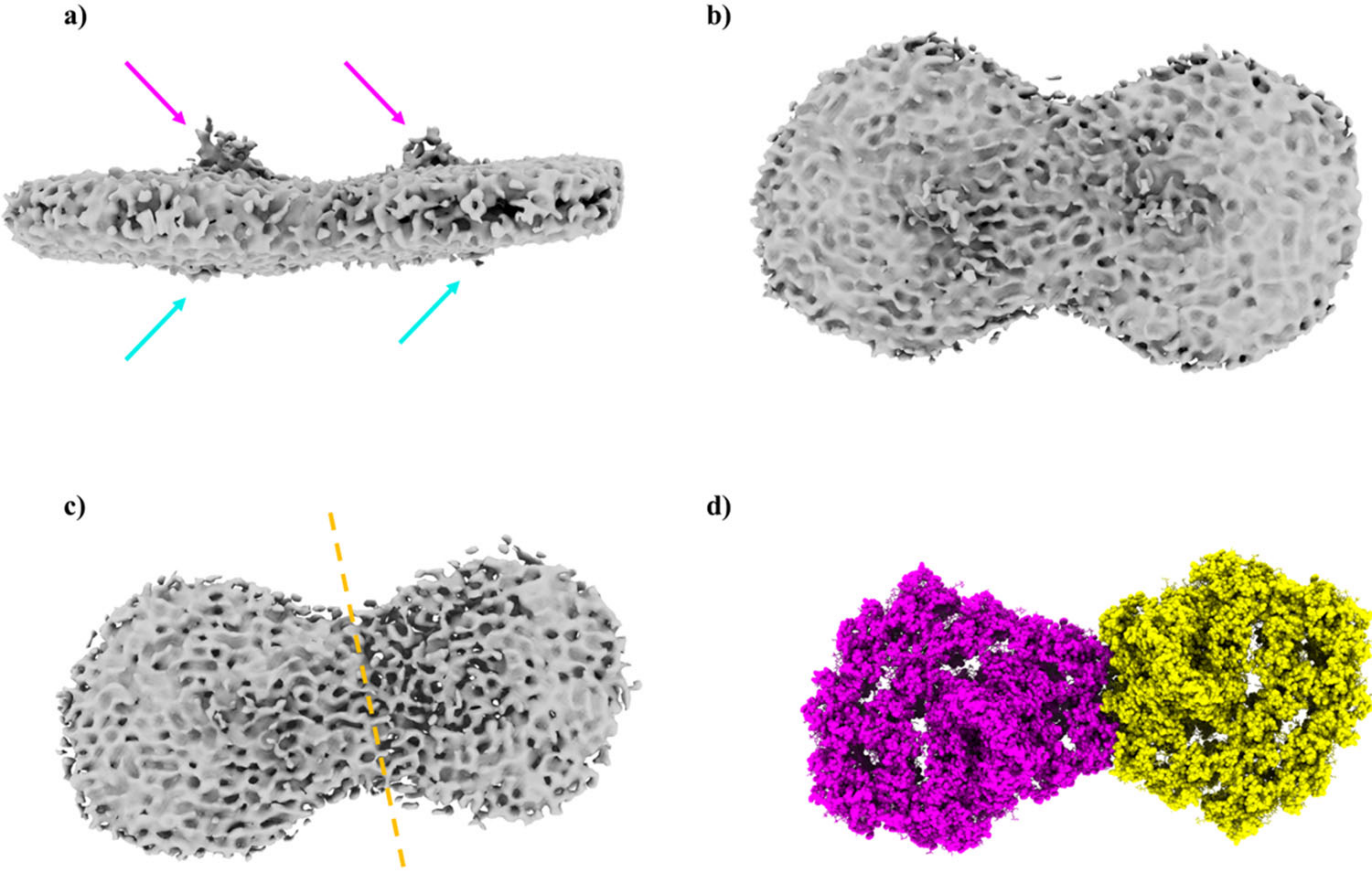
Low-resolution cryo-EM map of dimeric C. reinhardtii TSP4 PSI. **a** Membrane plane view of dimeric TSP4 PSI. The stromal subunits (PsaC, PsaD and PsaE) protrusion out of the membrane plan are marked with a magenta arrow, and the PsaF protrusion is marked with a cyan arrow. **b** Stromal view of the TSP4 dimer. **c** Stromal view with a lower map contour showing the borders of each PSI monomer, separated by a dashed orange line. **d** TSP4–10LHC (magenta) and TSP4–8LHC (yellow) as placed into the low-resolution map.

During the particle selection from the cryo-grids, a significant population of PSI dimer was recognised (Fig. 3). The identification of PsaD and PsaE on the stromal side and PsaF on the luminal side left little doubt that this was indeed a PSI dimer. Although this structure was not solved to a high resolution, a 17.1 Å 3-dimensional model was constructed, suggesting that the two PSI complexes - one containing 10 LHCs and the other 8 LHCs – are bound together where PsaH should be present. The dimer was formed by Lhca2 and Lhca9, which bound the PsaB subunits from both complexes, as well as PsaG from PSI-8LHC (Fig. 3). Alternatively, the dimer can be made by two PSI with 9 LHCs each. The presence of PSI with 8 LHCs has been reported for PSI from different algal preparations^18,19^, however it is unclear whether this form is naturally present in green algae or is observed due to the loss of LHC subunits during purification. *C. reinhardtii* dimerisation may be either a light-dependent adaptive mechanism, as previously postulated for the cyanobacterial PSI oligomers^29,30^, a compensation mechanism for the missing degraded PSII that may be related in an undisclosed fashion to cyclic eletron transport, or could also be a unique trait of TSP4, as this is the first report of PSI dimerisation in *C. reinhardtii* or any other eukaryote.

The modularity of PSI was demonstrated in several organisms, indicating that environmental stress may influence the composition of conserved membrane complexes such as PSI^25^. This flexibility was reflected in the frequent loss of green algae Lhca2 and Lhca9, and their LHCR equivalents in red algae^18,19,25,31–33^. It was worth noting that in the green lineage, the binding of the second LHC half-crescent belt was tighter than the Lhca2 and Lhca9 equivalents, and its absence in the PSI preparations may indicate its nonexistence in vivo^25^.

The strict conservation of the PSI core complex through its evolution from cyanobacteria to the higher plants has been widely discussed^5,29,34,35^. Over 90 chlorophyll molecules in the core complex have maintained nearly identical positions and orientations throughout 3.5 billion years of evolution. Similarly, 12 chlorophyll molecules in the various LHCs maintained almost identical positions from red through to green algae to the higher plants^36^. Remarkably, the position of the water molecules has remained fairly conserved during the long evolution of the complex^24^. The assignment of water molecules in the structures that were solved by cryo-EM was not as advanced as it is in X-ray crystallography. Therefore, it came as a surprise to detect a large number of densities that were reminiscent of electron densities that are routinely assigned as water molecules in crystal structures. This discovery allowed for the modelling of 337 water molecules in the structure. Remarkably, most of them were present in identical positions in plant PSI that was solved to 2.6 Å resolution^24^ (Supplementary Fig. 7a). Several of these water molecules were present in the vicinity of the light-induced electron transfer chain, which likely affected the oxidation-reduction processes within PSI. In all of the PSI structures that were reported for green lineage, a large aqueous channel, that is missing in cyanobacterial PSI structures^21,28^, approaching iron cluster FX in between PsaE and PsaB was present (Supplementary Fig. 7b). This “empty” space may easily accommodate a methyl viologen molecule (Supplementary Fig. 7c). Therefore, this cavity is now proposed to be the preferred methyl viologen electron-accepting site, based on the polypeptides and water molecules in the extended loop region. It is also plausible that methyl viologen binds at an alternative location in cyanobacterial PSI. This notion was supported by the observation that much higher concentrations of methyl viologen were required to inhibit cyanobacterial photosynthesis^37^. The cyanobacterial PSI structures showed that the extended PsaE loop blocked this water channel, thus preventing methyl viologen from binding in this site (Supplementary Fig. 7c).

Another group of water molecules occupied the fifth coordinate of chlorophyll magnesium. These water molecules should be present near all of the chlorophyll molecules that were not coordinated by the polypeptide backbone, the side chains or the phospholipids. Indeed, the *C. reinhardtii* PSI map densities clearly show this property, in accordance with several other chlorophyll-protein complexes that have been solved by X-ray crystallography^21,24,28,38,39^. Several of the magnesium coordinated water molecules were accompanied by a second water molecule, hence forming a slightly tilted L-shape of Mg-H_2_O-H_2_O (Fig. 2 and Supplementary Fig. 5). Nearly one-third of the chlorophyll b molecules (8/25) were populated by two water molecules, and the L-shape was more frequent in the LHC than PSI core (22 instances vs 11, respectively; Supplementary Table 1). The above observations have vindicated the water assignment in structures obtained at high-resolution cryo-EM.

First appearing in the evolution of cyanobacteria, close to 3 billion years ago, PSI is an ancient photochemical machine^35^. Given this long evolutionary history, it is remarkable that the core structure of PSI has remained virtually unchanged between cyanobacteria and the higher plants^25,27,38^. This structural conservation is related to the large network of light-harvesting pigments that are supported by the protein subunits, which restricts evolutionary plasticity. Within this network, the pigment–pigment distances and orientations are crucial to efficiently carry out the main function of the ETC, which is located in the centre of the complex. The most conserved region of PSI surrounds its three iron-sulphur clusters, which are located in PsaA, PsaB and PsaC. The first of these redox centres, FX, is conserved down to the surrounding water molecules^24^. This water shell extends into the highly hydrophobic membrane space (Fig. 2 and Supplementary Fig. 6a). The inclusion of any polar entity in this region involves a high-energy penalty.

The positions of the various co-factors in the two branches of the ETC were highly similar but the electron transfer to FX from the two-quinone donors, Q_A_ and Q_B_, differed by an order of magnitude^40^. The FX proximal water molecules were positioned within 10 Å of the cluster and this resulted in 24 water molecules that were identified in FX’s vicinity (Supplementary Fig. 7b). As noticed before, the position of several of these water molecules was conserved in the crystal structures of thermophilic and mesophilic cyanobacteria^21,24,28^. The spatial distribution of these conserved waters was not random; the conserved positions clearly lay at the ‘bottom’ (membrane buried) half of the FX water shell and they occupied the space separating the quinone electron donors and FX. The quinone that was bound by PsaA, Q_A_, was in close contact with a larger number of these conserved waters than the PsaB quinone Q_B_, and this may have accounted for the slower rate of electron transfer that was observed between this quinone and FX. It was not possible to accurately determine the orientation of these waters, as they were mainly coordinated by the interactions with the protein backbone. Their effect on the electron transport rate may stem directly from their interaction with Q_A_, for example, by raising its redox potential. Alternatively, they may affect the dielectric properties of the space between FX and Q_A_, which may be more polar than assumed in calculations, or perhaps they interact with FX directly, lowering the energy barrier that is associated with the electron transfer. Future experiments are needed to distinguish between these alternatives. The water molecules surrounding FX may be important to determine its redox potential and its capability to transfer its electrons to FA or water^41^, exemplifying that the potentials in these proteins are tuned, in part, by varying the access of solvent water to the local environment of the cluster.

These findings serve as proof for the validity of water molecule assignment, according to the cryo-EM density maps in the high-resolution structures. Thus, previously published PSI cryo-EM structures from *Dunaliella salina* and pea plants^25,42^ were revisited to seek unmodelled water molecules. Regardless of the method used for the structural determination, a significant number of bound water molecules were identified in the hydrophobic milieu surrounding the ETC, especially around FX (Supplementary Figs. 8–12).

**Fig. 4:**
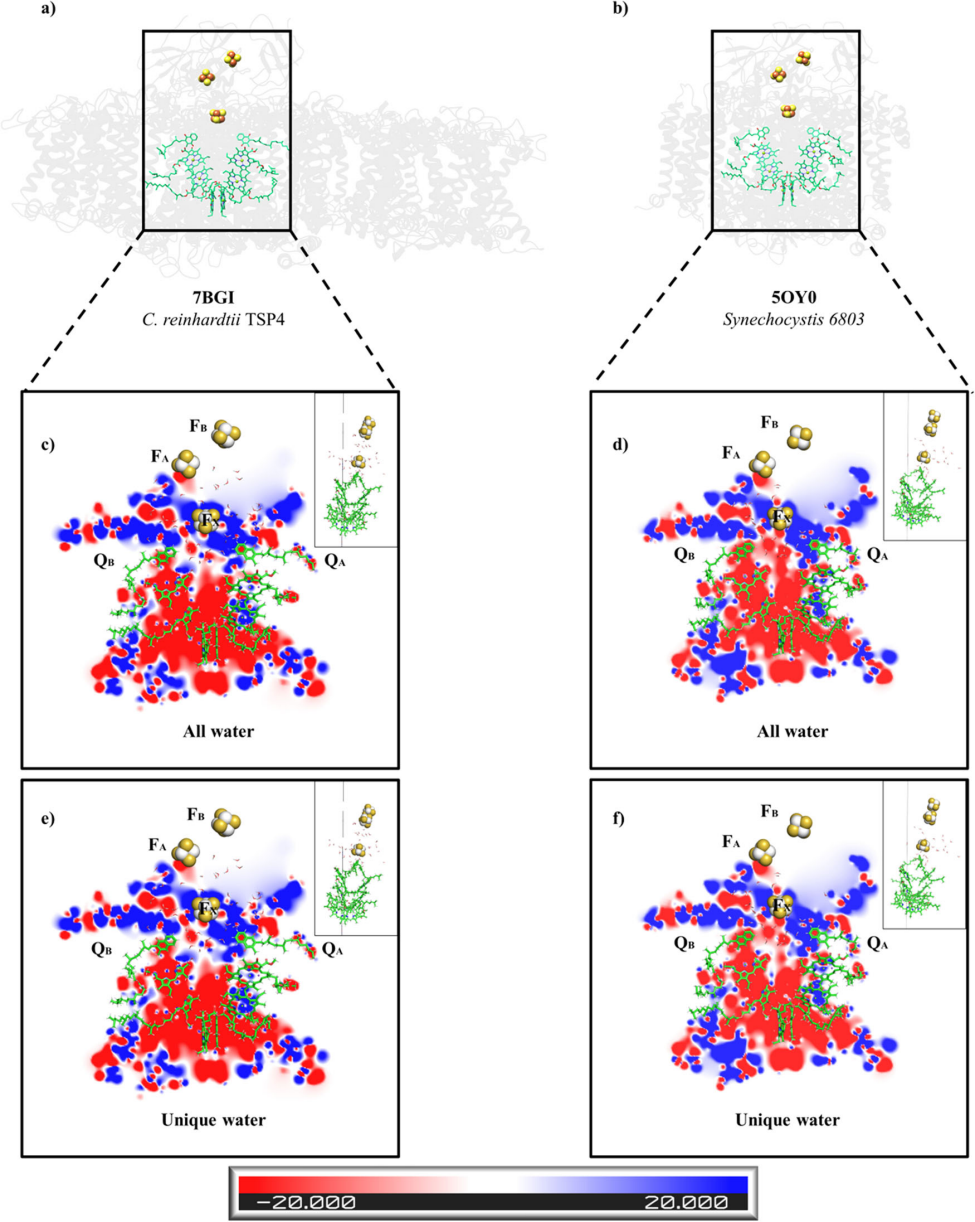
The electrostatic potentials of the C. reinhardtii TSP4 and Synechocystis PSI RC. The structure of *C. reinhardtii* TSP4 (**a**) and *Synechocystis* PSI (**b**) from a membrane plane view. For clarity, only the ligands of the proteins (chlorophylls, quinones and the iron-sulphur clusters) and the water molecules are presented and are marked with a rectangle. The electrostatic potentials were calculated on two conditions: with all of the water molecules found within a 10 Å radius of the FX of *C. reinhardtii* TSP4 (**c**), or *Synechocystis* (**d**), or the water molecules that constitute a unique subset to *C. reinhardtii* TSP4 (**e**), or *Synechocystis* (**f**). In each case presented hereafter (Supplementary Figs. 11–13), the electrostatic potentials are mapped onto three parallel slices, each located at a different position along the RC Y-axis (see the small image on the right side of each figure, showing the RC orthogonally, i.e. rotated 90° when compared with the main image). The negative potentials (0 *k*_*B*_T/e > Φ > −20 *k*_*B*_T/e) are red, the positive potentials (0 *k*_*B*_T/e < Φ < 20 *k*_*B*_T/e) are blue, and the neutral potentials are white (see the colour code at the bottom). Noticeable changes in electrostatic potential appear in the region between the quinones and FX, marked by black circles.

The ETC of all of the photosynthetic reaction centres exhibited a two-fold pseudosymmetry^5,34^. Numerous fast kinetics data when using a variety of techniques have revealed differences in the electron transfer rate in the two branches of the ETC^40,43–46^. These differences cannot be explained by the distance variances between the compounds that constitute the two branches. Parson et al.^47^ calculated the electrostatic interaction energies of the electron carriers with their surroundings in a photosynthetic bacterial reaction centre. They concluded that the electrostatic interactions with the proteins favoured the localisation of the positive charge of P^+^ on P_L_, one of the two bacteriochlorophylls molecules that make up the electron donor, accordingly making the L-branch preferable for charge separation when compared to the M-branch (the B and A branches in the heterodimeric reaction centres, respectively). Electron transfer studies carried out by electron paramagnetic resonance (EPR) and fourier-transform infrared spectroscopy (FTIR) on heterodimeric reaction centres concluded that an innate asymmetry exists in the P700 dimer^48^. This asymmetry is assignable to the chlorophyll a epimer called chlorophyll a’, situated in the PsaA side of P700. EPR and FTIR estimated the ratio of charge transfer from the PsaA (P_A_) or PsaB (P_B_) P700 chlorophylls^48–52^, exhibiting ratios varying from 1:1 to 1:6 in favour of P_B_. If electron transfer would go through the A branch, then P_A_, not P_B_, would need to initiate the charge transfer and would require that the positive charge retract from P_A_ to P_B_ to stabilise charge separation^48^. The bound water molecules were likely to influence the specific electrostatic calculations, especially when forming ordered structures that change water molecules’ chemical properties^53^, like the pentamer arrangement that was identified close to Q_B_ in TSP4 and *T. elongatus* (Supplementary Fig. 7c, black arrow). The electrostatic landscape in the vicinity of the ETC of *C. reinhardtii* and *Synechocystis* 6803 PSI is illustrated (Fig. 4; Supplementary Figs. 13–15 and Supplementary Movie 1–7), including or excluding the water molecules from both structures. The most apparent difference was in the electrostatic potential of both branches, where the A-branch enclosed a combination of electro-negative and electro-positive regions, whereas the B-branch was almost exclusively electro-negative. By removing the water molecules, a nearly identical electrostatic tendency was observed comparing *C. reinhardtii* to *Synechocystis* (Supplementary Fig. 15). The vicinity of P700 exhibited a symmetric negative potential in both of the B and A branches. The region surrounding FX was largely positive but patches of an electrostatic negative potential were protruding, preferentially at the B-branch. The large positive potential, which extended to the area between FX and Q_A_, might slow down the electron transfer between the two, by trapping the electrons in the potential well. This would support electron transfer via the B-branch, which did not contain the electropositive potential well versus the A-branch, as observed by Joliot and his colleagues^40^. Similar analyses, which were performed with cyanobacterial PSI, revealed the largely symmetric appearance of the negative patches of both branches (Fig. 4; Supplementary Fig. 13 and Supplementary Movie 1–7). Indeed, it has been reported that in cyanobacteria, the preferential branch, if any, is the A-branch^44,46^. The conserved water molecules appeared to be the main contributors to the different electrostatics that were seen in both branches (Fig. 4; Supplementary Figs. 13–15 and Supplementary Movie 1–7).

The cryo-EM revolution helped to surmount the obstacle that was posed by x-ray crystallography, by studying the structure of the photosynthetic membrane complexes, as evidenced by the diversity of structures that have been published in recent years^54^. The quality and assortment of the near-atomic resolution of PSI have previously revealed that not only the polypeptide sequence, but the ligands are highly conserved in their location and distribution. The high-resolution *C. reinhardtii* TSP4-LHC10 structure uncovered that a structured network of water molecules was also present in the PSI core complex, and the LHCs alike, were conserved in the cyanobacteria, in the green alga and the higher plants. These membrane-embedded water molecules have been proposed to serve a dual role, as ligands for all of the uncoordinated chlorophylls, as well as for the electrostatic modulators of the ETC. The analyses have implied that the asymmetric water distribution in proximity to Q_A_, Q_B_ and FX might affect the charge transfer in the A and B branches, making the latter, the preferable branch for the electron transfer. The data that was collected contained two additional PSI conformations, TSP4–8LHC, previously described in *C. reinhardtii*^18,19^, and a small population of PSI dimers, which is the first report of PSI oligomerisation in eukaryotes.

## Methods

### PSI purification

*C. reinhardtii* strain TSP4xpgr5 was grown in 10 liter TAP media^55^ for 3 days at 25 °C under continuous illumination (50 µmol m^−2^ s^−1^) to a cell density of OD_730_ = 0.82. The culture was harvested by centrifugation at 1000 × *g* for 5 min, resuspended in fresh 10 L of TAP media preheated to 37 °C and left to adapt for 14 h at 37 °C under continuous illumination (50 µmol m^−2^ s^−1^). Then, the culture was harvested by centrifugation (Sorvall F10S 4 × 1000 LEX; 12,160 × *g*; 4 min) for thylakoid membranes preparation. The pellet was resuspended and washed in 200 ml STN buffer (20 mM Tricine-Tris pH 8.0; 15 mM NaCl; 0.3 M sucrose), pelleted again by centrifugation (Sorvall rotor SS-34; 7650 × *g*; 4 min) and resuspended in 50 ml of ice-cold STN buffer. The cells were broken using a French press (Avestin® EmulsiFlex-C3 electric motor; 6 times; 1500 psi). Unbroken cells and starch were removed by centrifugation at (Sorvall rotor SS-34; 17,200 × *g*; 10 min) and discarded. The supernatant was centrifuged at higher speed (Beckman rotor Ti-70; 45,000 rpm; 2 h) to recover the thylakoid membranes. The pelleted membranes were resuspended and washed in 160 ml STN buffer supplemented with 150 mM NaCl, pelleted again by centrifugation at 207,000 × *g* for 1 h and finally the pellet was resuspended in 20 ml STN buffer. The membranes were diluted to a chlorophyll concentration of 1 mg ml^−1^, supplemented with 1.5 % of **α**DM (n-Decyl-**α**-d-Maltopyranoside) and incubated on ice for 30 min. Insoluble material was removed by centrifugation at 250,000 × *g* for 1 h. The supernatant was centrifuged at 430,000 × *g* for 1 h. The pellet was resuspended in 10 ml TN buffer (20 mM Tricine-Tris pH 8.0; 15 mM NaCl) and diluted to a chlorophyll concentration of 1 mg ml^−1^. The sample was loaded on a 50 ml DEAE anion-exchange column that was previously equilibrated with TN buffer containing 0.2 % **α**-DM. The column was washed with the same buffer containing 50 mM NaCl and the particles were eluted with the same buffer containing 200 mM NaCl. The elution fractions with the highest chlorophyll concentration were loaded on sucrose gradients (TN buffer; 15–50% sucrose; 0.1% **α**-DM) and centrifuged (Beckman rotor SW-60; 309,600 × *g*; 14 h). The darkest band was collected (Supplementary Fig. 16a), PEG precipitated^28^ and resuspended in a buffer containing 20 mM Tricine (pH 8.0) to a chlorophyll concentration of 3.7 mg ml^−1^. SDS-PAGE of the heavy band is shown in Supplementary Fig. 16b.

### Cryo-EM data collection and processing

The resulting solution of purified PSI (3 μl) was applied onto glow-discharged holey carbon grids (Cu Quantifoil R1.2/1.3) before vitrifying using a Vitrobot FEI (3 s blot at 4 °C and 100% humidity). The images were collected using a 300 kV FEI Titan Krios electron microscope, with a slit width of 20 eV on a GIF-Quantum energy filter, at the ESRF cryo facility, Grenoble, France. A Gatan K3-Summit detector was used in counting mode at a magnification of 165,000 (yielding a pixel size of 0.827 Å), with a total dose of 46.8 e Å − 2. EPU was used to collect a total of 12,418 images, which were dose-fractionated into 40 video frames, with defocus values of 0.9–3.0 μm at increments of 0.3 μm. The collected micrographs were motion-corrected and dose-weighted using MotionCor2^56^. The contrast transfer function parameters were estimated using CtfFind v.4.1^57^. A total of 1,208,518 particles were picked using LoG reference-free picking in RELION3^58^. The picked particles were processed for reference-free two-dimensional (2D) averaging. After several rounds of 2D classification, which resulted in 329,115 particles, an initial model was generated using RELION3. A total of 101,572 particles (PSI-10LHC), 17,311 (PSI-8LHC) or 5707 (PSI-dimer) were pooled together and processed for 3D homogeneous refinement and postprocessing using RELION. To improve the map density around Lhca2 and Lhca9 in PSI-10LHC, focused refinement was performed and the resulting maps were merged using combine_focused_maps^59^. The reported resolutions were based on a gold-standard refinement, applying the 0.143 criterion on the FSC between the reconstructed half-maps. (Supplementary Figs, 1 and 2).

### Model building

To generate the PSI-10LHC, the cryo-EM structure of *C. reinhardtii* PSI model PDB 6JO5 was selected. This model was fitted onto the cryo-EM density map, and manually rebuilt using Coot^60^. Stereochemical refinement was performed using phenix.real_space_refine in the PHENIX suite^59^. The final model was validated using MolProbity^61^. The refinement statistics are provided in Table 1. Local resolution was determined using RELION local resolution^58^, and the figures were generated using PyMOL (Schrödinger), UCSF Chimera^62^ and ChimeraX^63^.

### Kinetics of light-induced P700 oxidation and reduction

The WT and the TSP4 strains were grown in 1 L liquid TAP + medium^55^ (Tris, Acetate, phosphate at pH 7.0), at 25 °C, while shaking, under continuous illumination (30–45 μE). For each of the strains, the cultures were divided equally to three conditions, TAP + was added to reach final volume of 1 L. Initial inoculations and dilutions were aimed to reach a logarithmic growth stage (measured O.D at 730 nm of 0.2–0.7) at the time of the measurement. On the day of the measurements, 500 ml of the cultures were concentrated by Sorvall F10S 4 × 1000 LEX at 6800×*g* for 8 min. The pellet was homogenised into 5 ml using the medium extracted from the same culture. The chlorophyll content of the concentrated cultures was measured, and all samples were diluted (using their respective extracted mediums) to reach the same balanced chlorophyll content of 0.1 mg/ml. The balanced samples were well mixed and placed in a crystal cuvette to be measured for light-induced oxidation of P700 to P700^+^ and re-reduction of P700^+^ to P700. This was done by recording absorption changes at 705 nm using a Joliot-Type Spectrophotometer-10^25^ (JTS). P700^+^ reduction kinetics analysis was performed on the two strains in three conditions: first the strains were grown for 24 h in the permissive temperature of 25 °C, afterwards the strains were grown at the non-permissive temperature of 37 °C for another 24 h. Last, heat-treated samples were transferred to recovery for another 24 h at the permissive temperature of 25 °C. The strains reactions to varying temperatures were measured in the absence and presence of electron transporters^64^. Background noise had to be adjusted manually per each measurement. During the measurement, samples were subjected to a sequence of 35 short light flashes at 940 µE/m²/s for 0.1 second with a pump wavelength of 630 nm, in order to induce photo-oxidation of P700 to P700^+^. The signals were detected during short dark phases immediately after each light flash with a probe at 705 nm with intensities varying 3–10 µE/m²/s. P700^+^ re-reduction signals were recorded in the following 8 s, demonstrating the rate of PSI kinetics in the dark.

### Electrostatic potential calculation

The potential maps were calculated using the Adaptive Poisson-Boltzmann Solver^65^ (APBS), with protein and solvent dielectrics of 2 and 78, respectively, ionic concentration of 0.1 M, and by using grid focusing. Partial charges and atomic radii were assigned to the protein using the PARSE set^66^, and to hetero-molecules (chlorophylls, quinones and iron-sulphur clusters) using the Electronegativity Equalization Method^67^ (EEM).

### Reporting summary

Further information on research design is available in the Nature Research Reporting Summary linked to this article.

## Supplementary information

Supplementary Information

Description of Additional Supplementary Files

Supplementary Movie 1

Supplementary Movie 2

Supplementary Movie 3

Supplementary Movie 4

Supplementary Movie 5

Supplementary Movie 6

Supplementary Movie 7

Reporting Summary

## Data Availability

The atomic coordinates have been deposited in the Protein Data Bank, with accession code 7BGI (PSI-10LHC), 7BLX (PSI-8LHC) and 7O01 (PSI-dimer). The cryo-EM maps have been deposited in the Electron Microscopy Data Bank, with accession codes EMD-12180 (PSI-10LHC), EMD-12227 (PSI-8LHC) and EMD-12672 (PSI-dimer). Any remaining information can be obtained from the corresponding author upon reasonable request.
