# Supplementary material for: Dimeric and high-resolution structures of Chlamydomonas Photosystem I from a temperature-sensitive Photosystem II mutant
Source: Commun Biol. 2021 Dec 9;4:1380. doi: 10.1038/s42003-021-02911-7 (PMC8660910; doi:10.1038/s42003-021-02911-7)
Supplement: Supplementary file 1 — Supplementary Information [file 42003_2021_2911_MOESM1_ESM.pdf]

Supplementary Information

Dimeric and high-resolution structures of Chlamydomonas Photosystem I from a temperature-sensitive Photosystem II mutant

Ido Caspy[1], Tom Schwartz[1]*, Vinzenz Bayro-Kaiser[1], Mariia Fadeeva[1], Amit Kessel[1], Nir Ben-Tal[1], and Nathan Nelson[1]***

[1]Department of Biochemistry and Molecular Biology,
The George S. Wise Faculty of Life Sciences, Tel Aviv University, Tel Aviv, 69978, Israel

**Equally contributed to the experimental work presented.*

****Corresponding author**: nelson@tauex.tau.ac.il

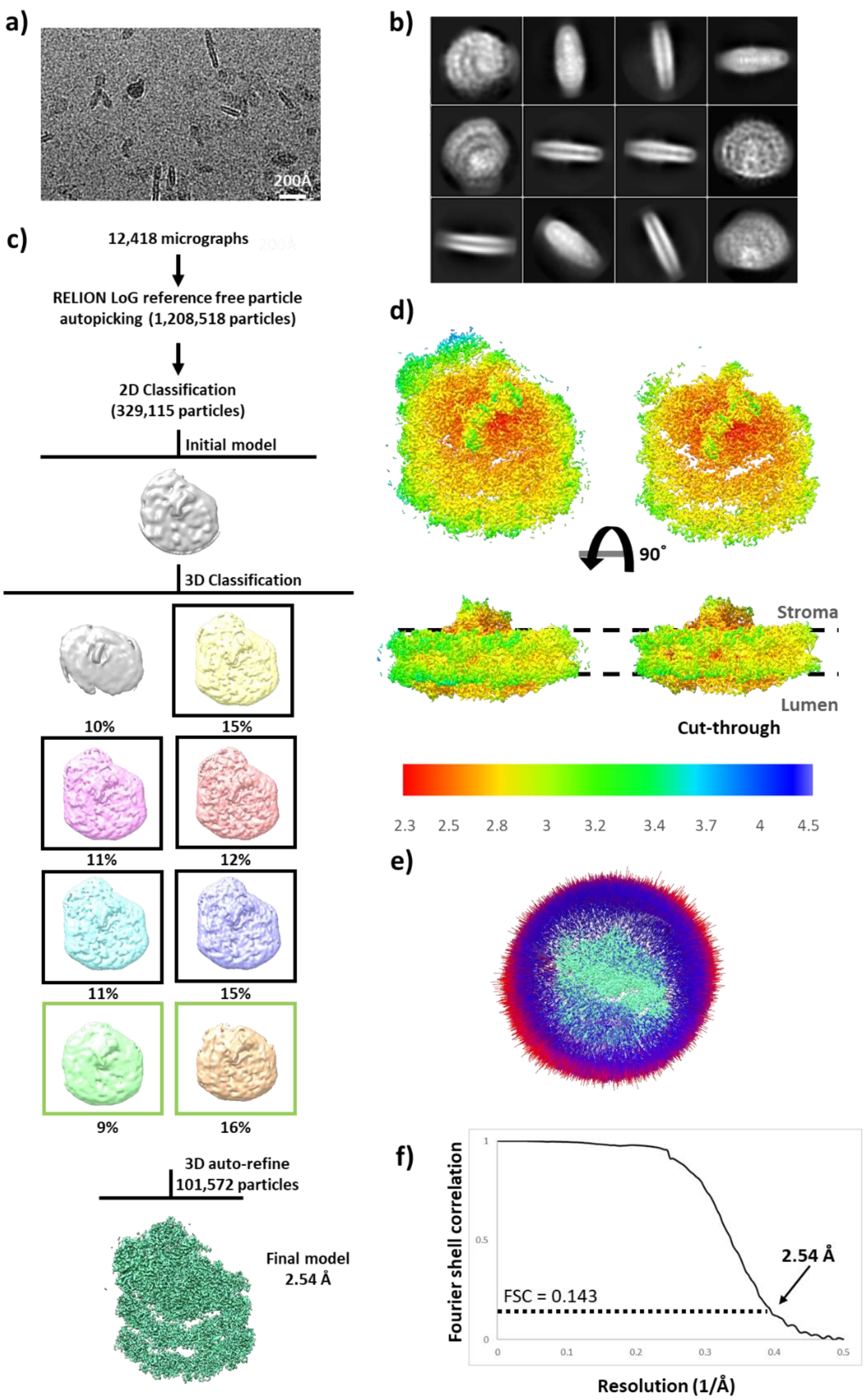

Supplementary Figure 1. Cryo-EM data collection and processing of TSP4-10LHC. a) A zoomed-in view on a TSP4 micrograph. Scale bar size is 200 Å. b) Representing class averages after 2D classification. c) Cryo-EM data processing workflow. d) Local resolution of TSP4-10LHC (left) and cut-through map (right). e) Angular distribution of TSP4-10LHC. f) Fourier shell correlation (FSC) RELION postprocessing result marks a 2.54 Å resolution based on the 0.143 gold standard.

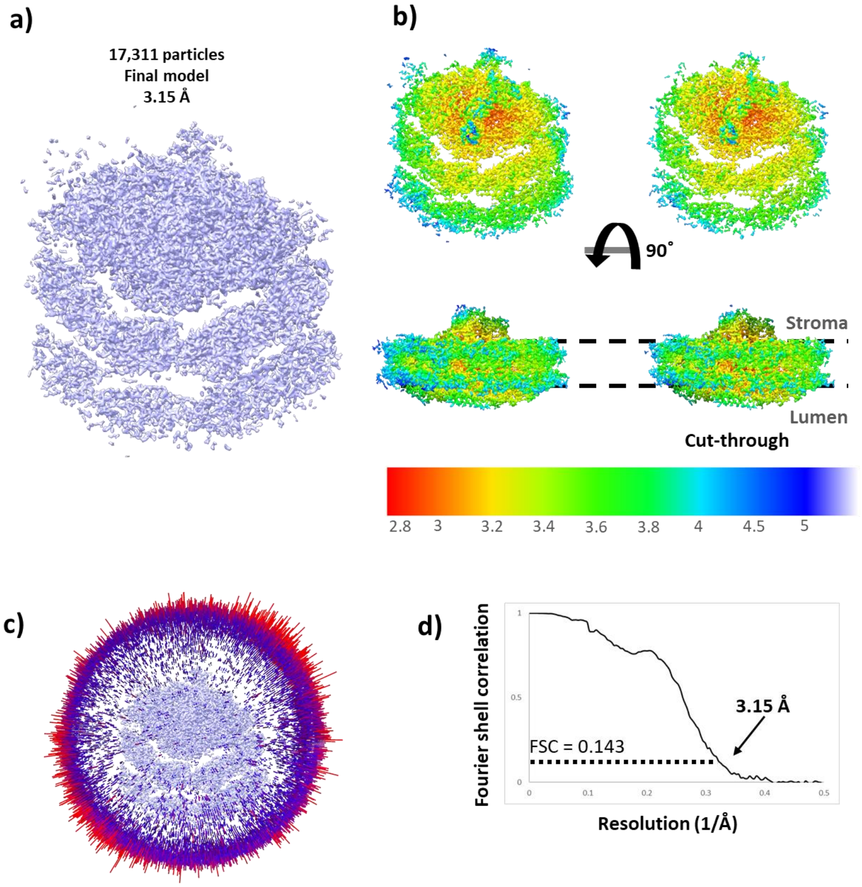

Supplementary Figure 2. Cryo-EM data collection and processing of TSP4-8LHC. a) The TSP4-8LHC final map after sharpening. b) Local resolution of TSP4-8LHC (left) and cut-through map (right). c) Angular distribution of TSP4-8LHC. d) Fourier shell correlation (FSC) RELION postprocessing result marks a 3.15 Å resolution based on the 0.143 gold standard.

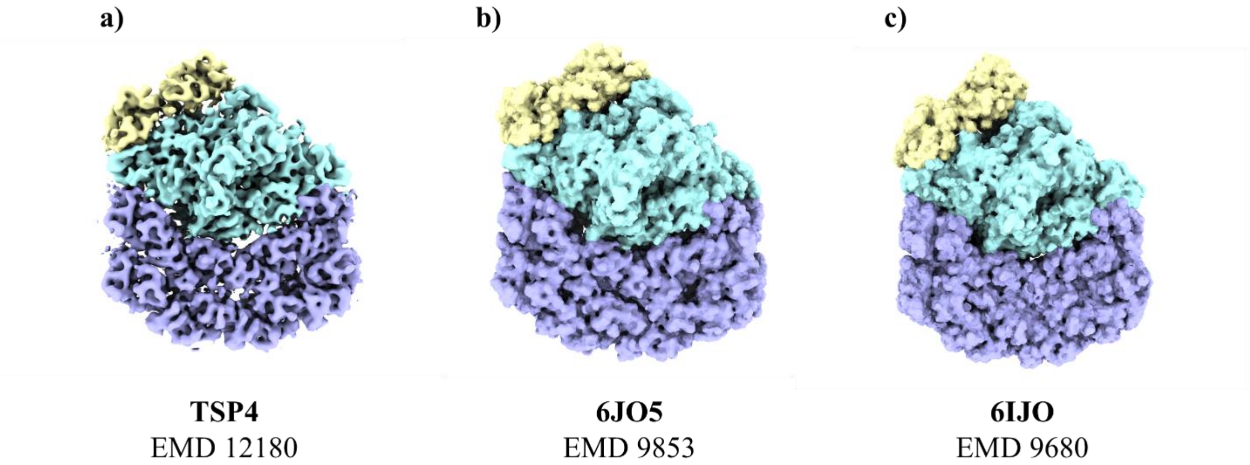

Supplementary Figure 3. The map quality of TSP4-10LHC when compared to the previous *C. reinhardtii* PSI. a) 2.54 Å TSP4-10LHC (PDBID 7BGI; EMD 12180). b) 2.90 Å *C. reinhardtii* PSI[19] (PDBIB 6JO5; EMD 9853). c) 3.30 Å *C. reinhardtii* PSI[18] (PDBIB 6IJO; EMD 9680). All of the maps were low-pass filtered to 6 Å, with gaussian filtered in Chimera[62], and placed at a contour of 0.0045. The core complex is coloured in cyan, the LHC belts in blue, and the Lhca2-Lhca9 dimer in yellow.

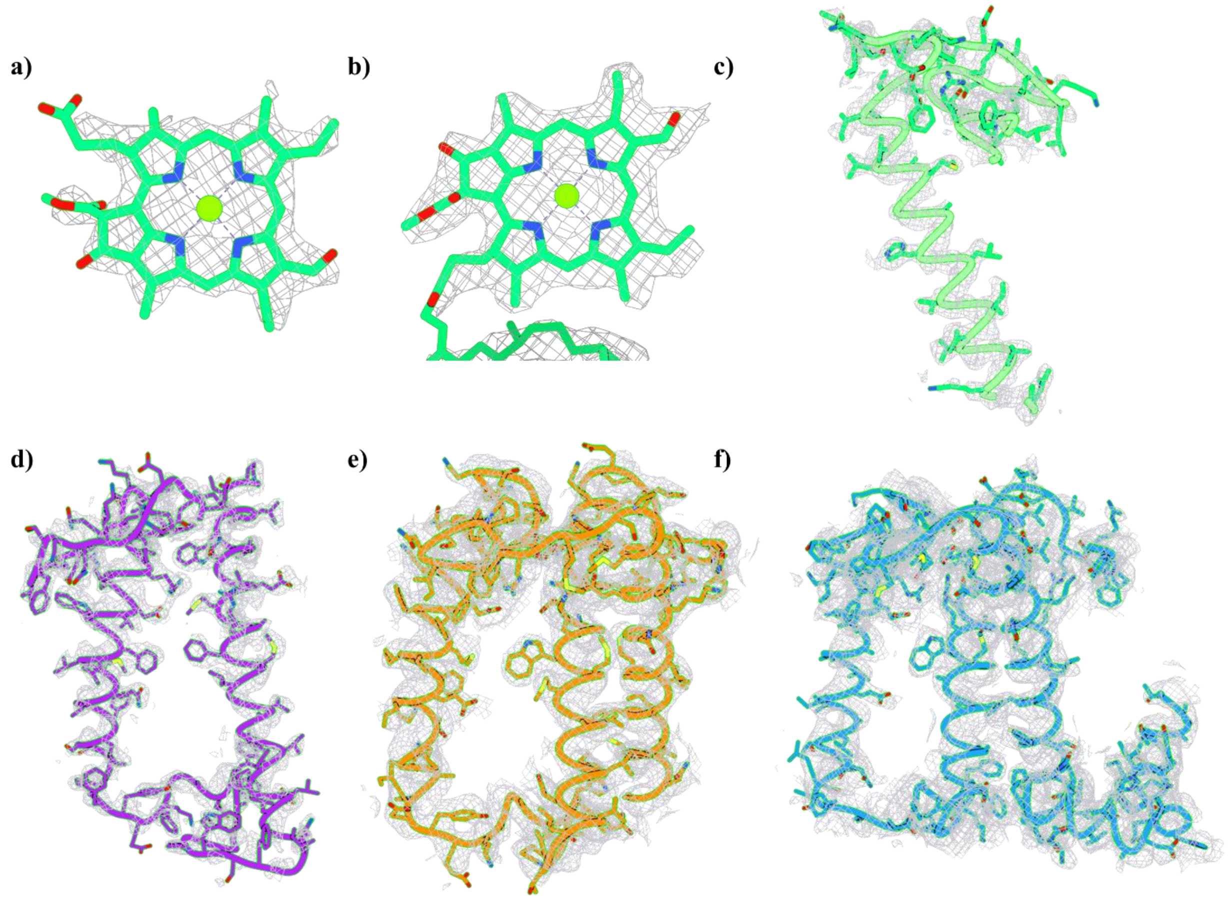

Supplementary Figure 4. Map densities of TSP4 PSI. a) Lhca1’ chlorophyll b 613 with the cryo-EM map at 3.5σ contour. b) Lhca8 chlorophyll b 613 with the cryo-EM map at 3.5σ contour. c) PsaK loop and second helix, residues 48-112, with with the cryo-EM map at 3σ contour. d) Lhca4 loop and two helices, residues 90-190, with with the cryo-EM map at 3σ contour. e) Lhca9 loop and three helices, residues 49-176, with with the cryo-EM map at 3σ contour. f) Lhca2 loop and three helices, residues 49-210, with with the cryo-EM map at 3σ contour.

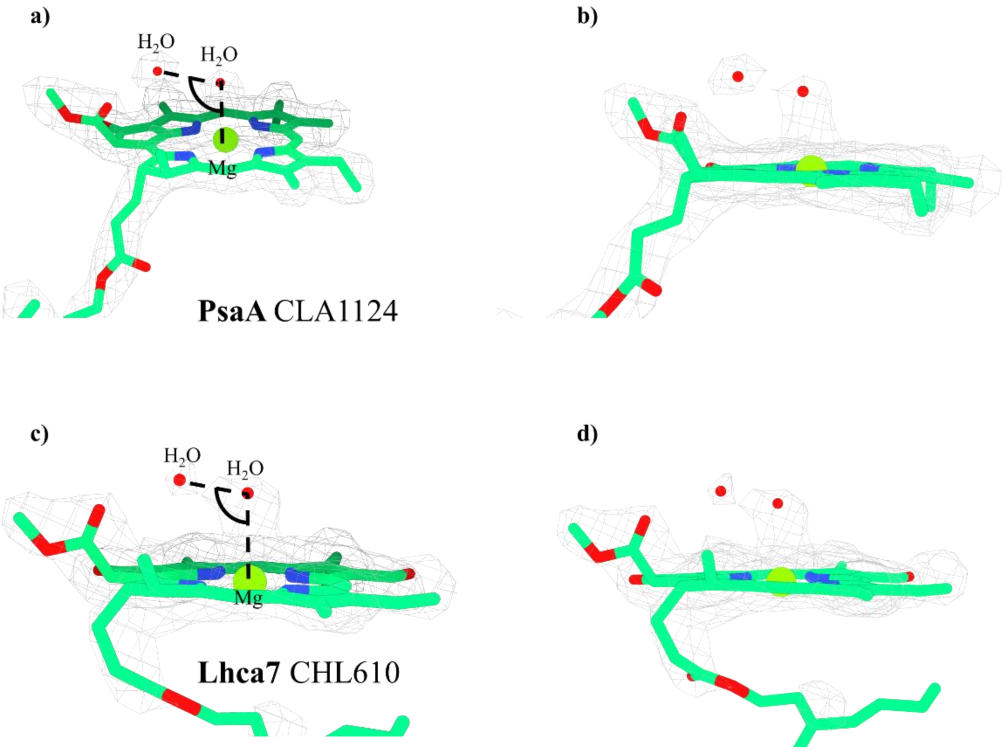

Supplementary Figure 5. The H_2O-H_2O-Mg chlorophyll coordination in TSP4 PSI. a) PsaA chlorophyll a 1124 coordinated by two water molecules, the dashed line shows the L shape formed. b) Porphyrin plane image of chlorophyll a, with the cryo-EM map at 4σ contour surrounding the chlorophyll showing the water molecule density. c) Lhca7 chlorophyll b 610 coordinated by two water molecules, the dashed line shows the L shape formed. d) Porphyrin plane image of chlorophyll b, with the cryo-EM map at 4σ contour surrounding the chlorophyll showing the water molecule density. The carbons are coloured spring-green, the nitrogens in blue, the oxygens in red, and magnesium as bright-green spheres.

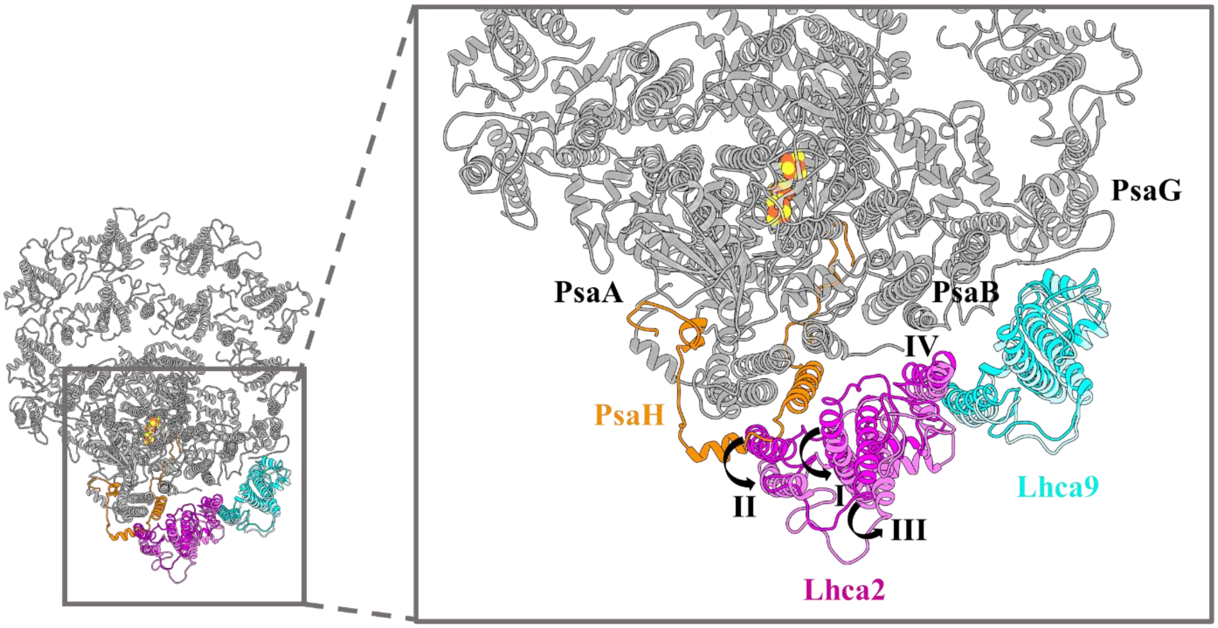

Supplementary Figure 6. Lhca2-9 movement caused by the PsaH insertion in *C. reinhardtii* PSI-LHCI-LHCII. A zoom-in on the Lhca2-Lhca9 dimer of TSP4 and in *C. reinhardtii* PSI-LHCI-LHCII[26] (PDBID 7D0J). The insertion of PsaH caused Lhca2 helices I, II, and III to move away from PsaB, while Lhca9 also moved but to a lesser extent. PsaH is coloured orange, TSP4 Lhca2 in magenta, 7D0J Lhca2 in pink, TSP4 Lhca9 in cyan, and 7D0J in light-blue, with the remaining PSI subunits in grey.

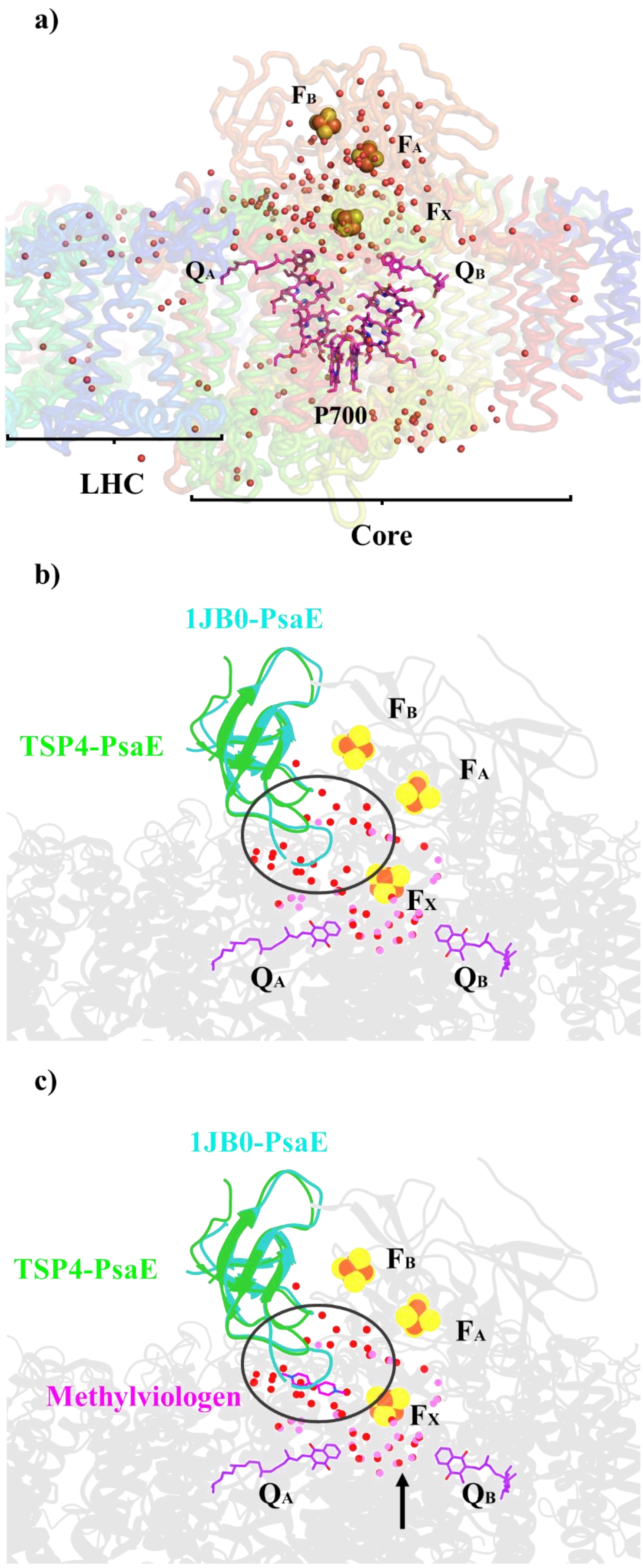
a)
F_B
F_A
F_X
Q_A
Q_B
P700
LHC
Core
b)
1JB0-PsaE
TSP4-PsaE
F_B
F_A
F_X
Q_A
Q_B
c)
1JB0-PsaE
TSP4-PsaE
F_B
F_A
Methylviologen
F_X
Q_A
Q_B

Supplementary Figure 7. Water common to the plant and the TSP4 PSI structures, and the effect of the PsaE loop on the water distribution in cyanobacteria and *C. reinhardtii*. a) The conserved water molecules between TSP4-10LHC and the plant PSI[24] (PDBID 5L8R). The largest clusters are found near the ETC and in the LHCs. The ETC components are coloured purple, the iron-sulphur clusters in yellow-brown, and the water molecules as red spheres. b) Superposition of TSP4 and *Thermosynechococcus elongatus* PSI[21] (PDBID 1JB0) when comparing PsaE and the water molecules close to FX, FA, FB, and the quinones. The extended PsaE loop of 1JB0 and the cavity filled with water molecules and its absence in TSP4 are encircled. The TSP4 water molecules are coloured red, the 1JB0 water molecules are coloured orchid, TSP4 PsaE is coloured green, 1JB0 PsaE in cyan, and the quinones in purple. c) The proposed methyl viologen binding site is the same cavity as encircled in (b), adjacent to FX. The 1JB0 extended PsaE loop prevents methyl viologen access to FX. Black arrow shows the water pentamer structure near Q_B.

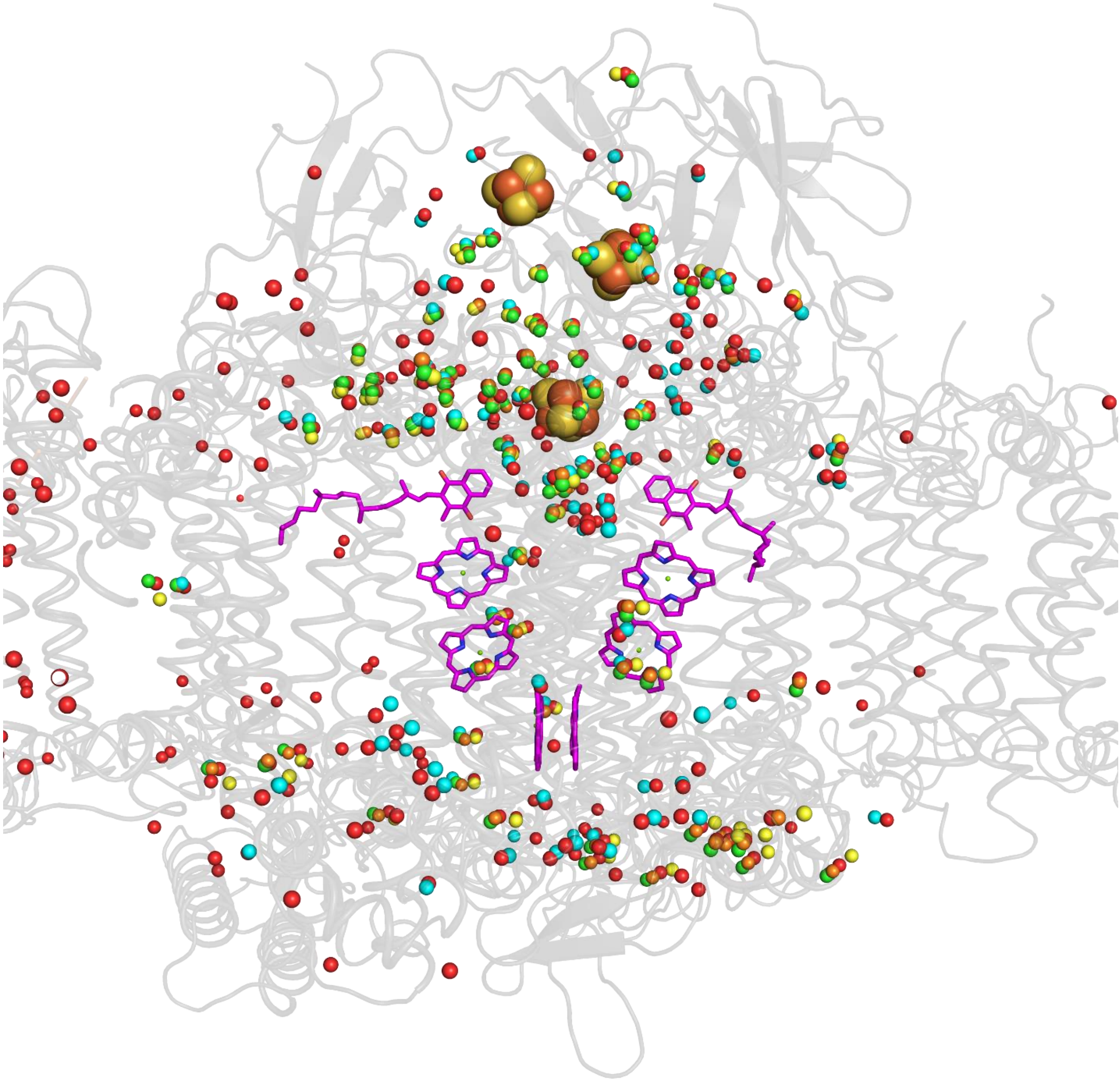

Supplementary Figure 8. The common water in PSI of TSP4, plant PSI-Fd, *Chlorella ohadii*, *Dunaliella salina* and *Thermosynechococcus elongatus*. Water molecules from each organism are shown as spheres. TSP4 water are colored red, plant PSI-Fd in green, *D. salina* in orange, *C. ohadii* in yellow and *T. elongatus* in cyan. PSI polypeptide is shown as a grey cartoon, ETC components are shown as magenta sticks and iron-sulphur clusters as yellow-brown spheres.

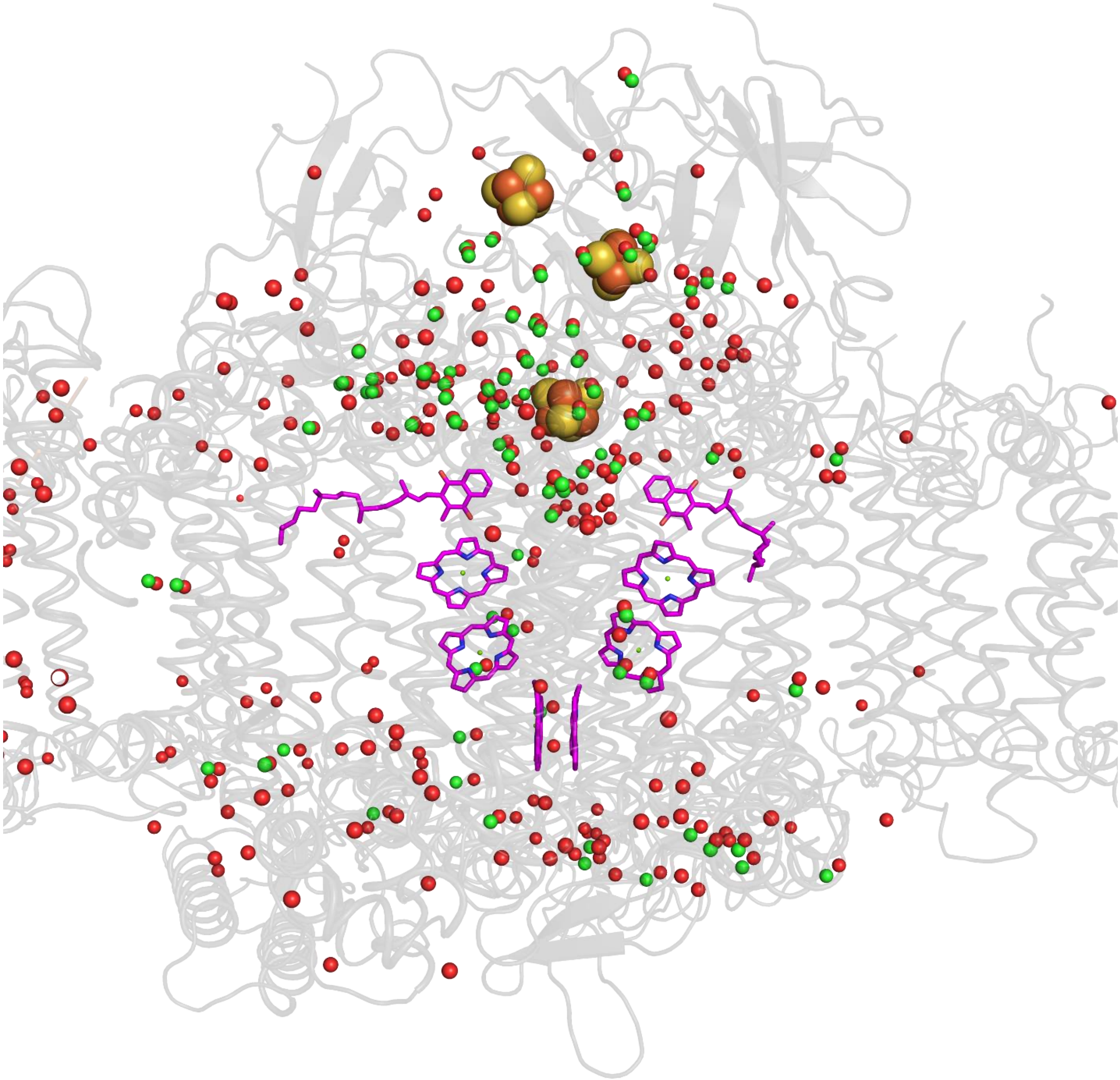

Supplementary Figure 9. The common water of TSP4 and plant PSI-Fd. TSP4-10LHC water molecules are shown as red spheres and PSI-Fd[42] (PDBID 6YAC) water molecules are shown as green spheres. PSI polypeptides and ETC components are coloured as in Supplementary Figure 8.

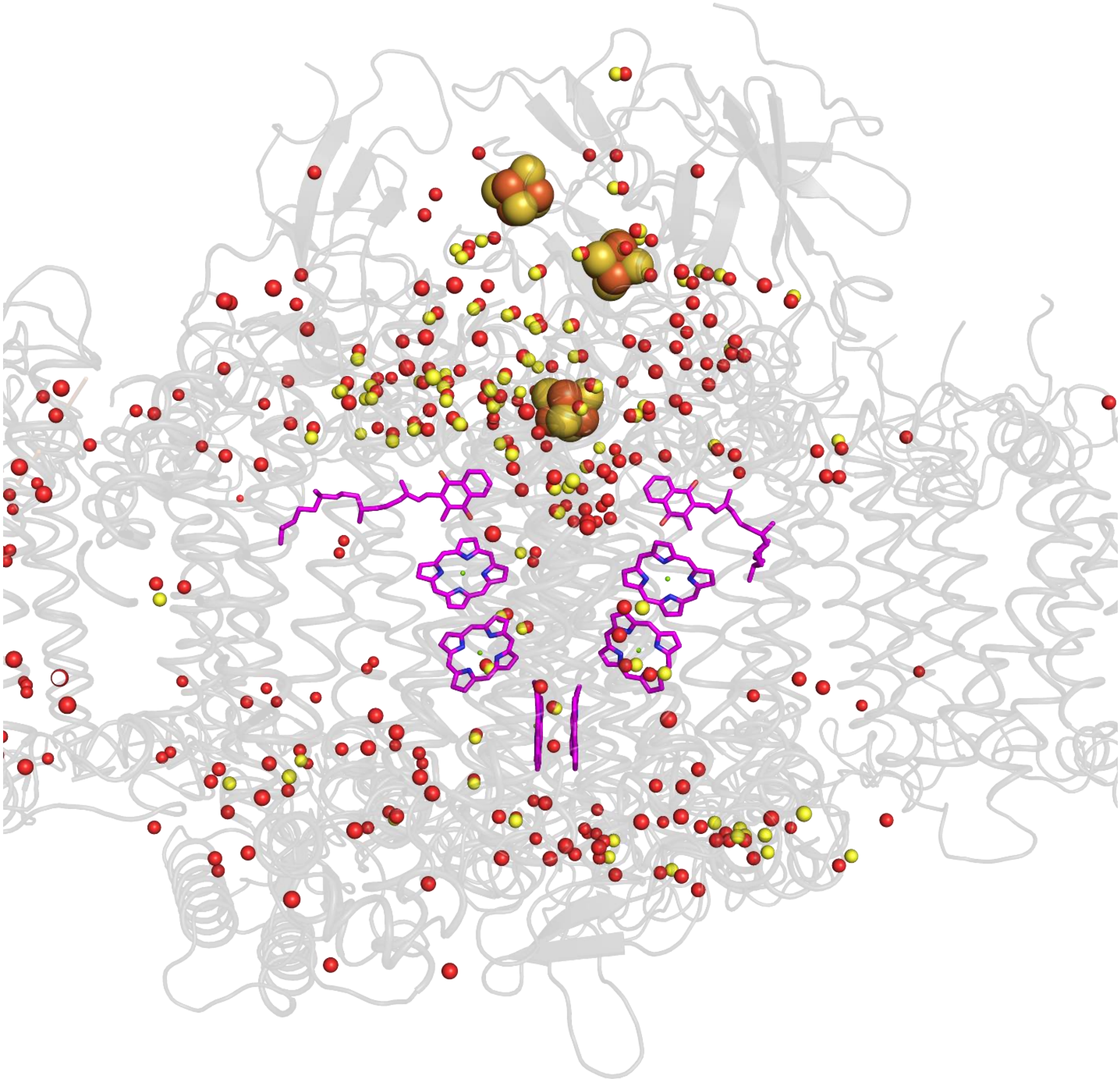

Supplementary Figure 10. The common water of TSP4 and *Chlorella ohadii* PSI. TSP4-10LHC water molecules are shown as red spheres and *C. ohadii* PSI (PDBID 6ZZX; Caspy et al., accepted for publication) are shown as yellow spheres. PSI polypeptides and ETC components are coloured as in Supplementary Figure 8.

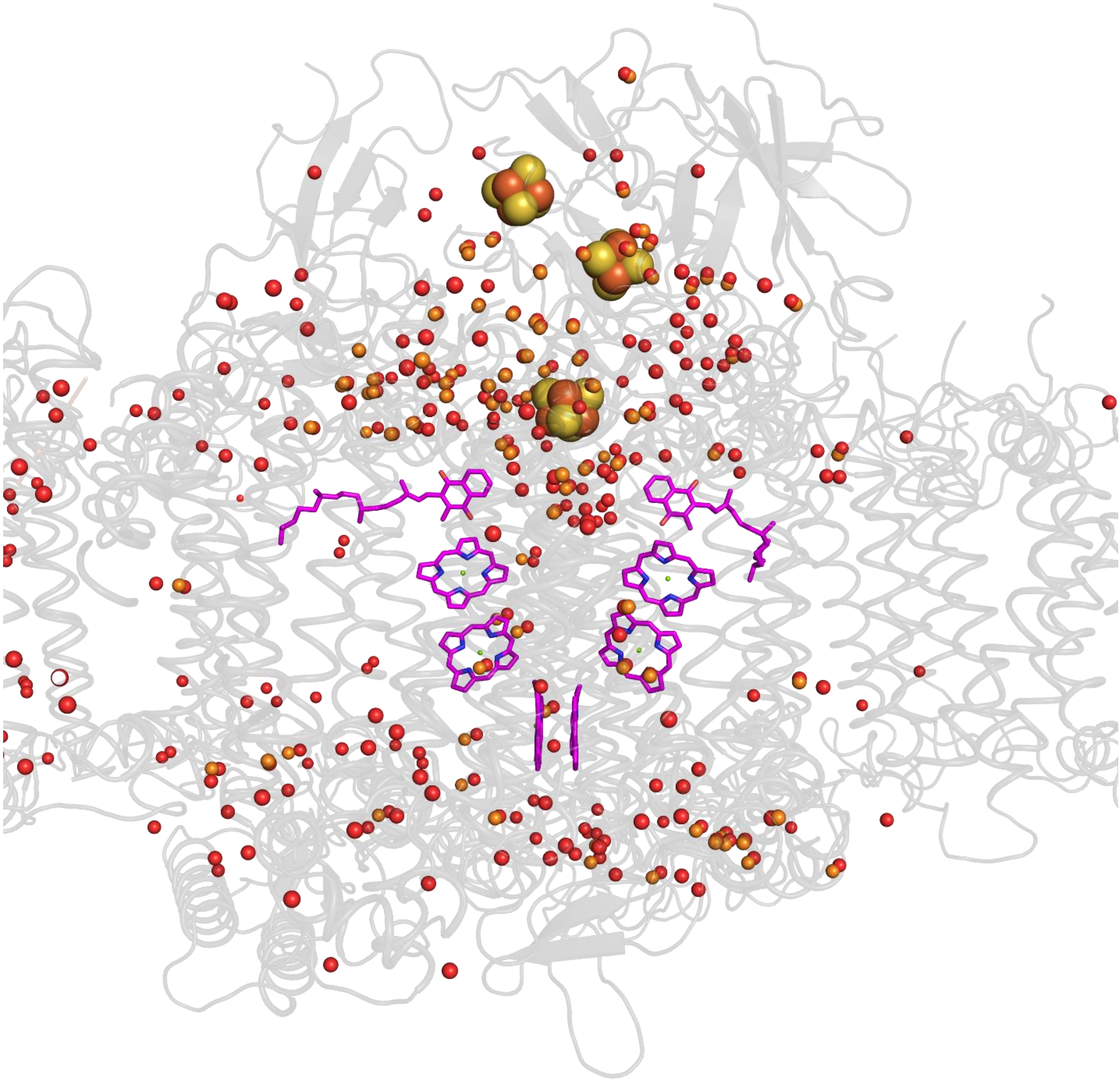

Supplementary Figure 11. The common water of TSP4 and *Dunaliella salina* PSI. TSP4-10LHC water molecules are shown as red spheres and *D. salina* PSI[25] (PDBID 6SL5) are shown as orange spheres. PSI polypeptides and ETC components are coloured as in Supplementary Figure 8.

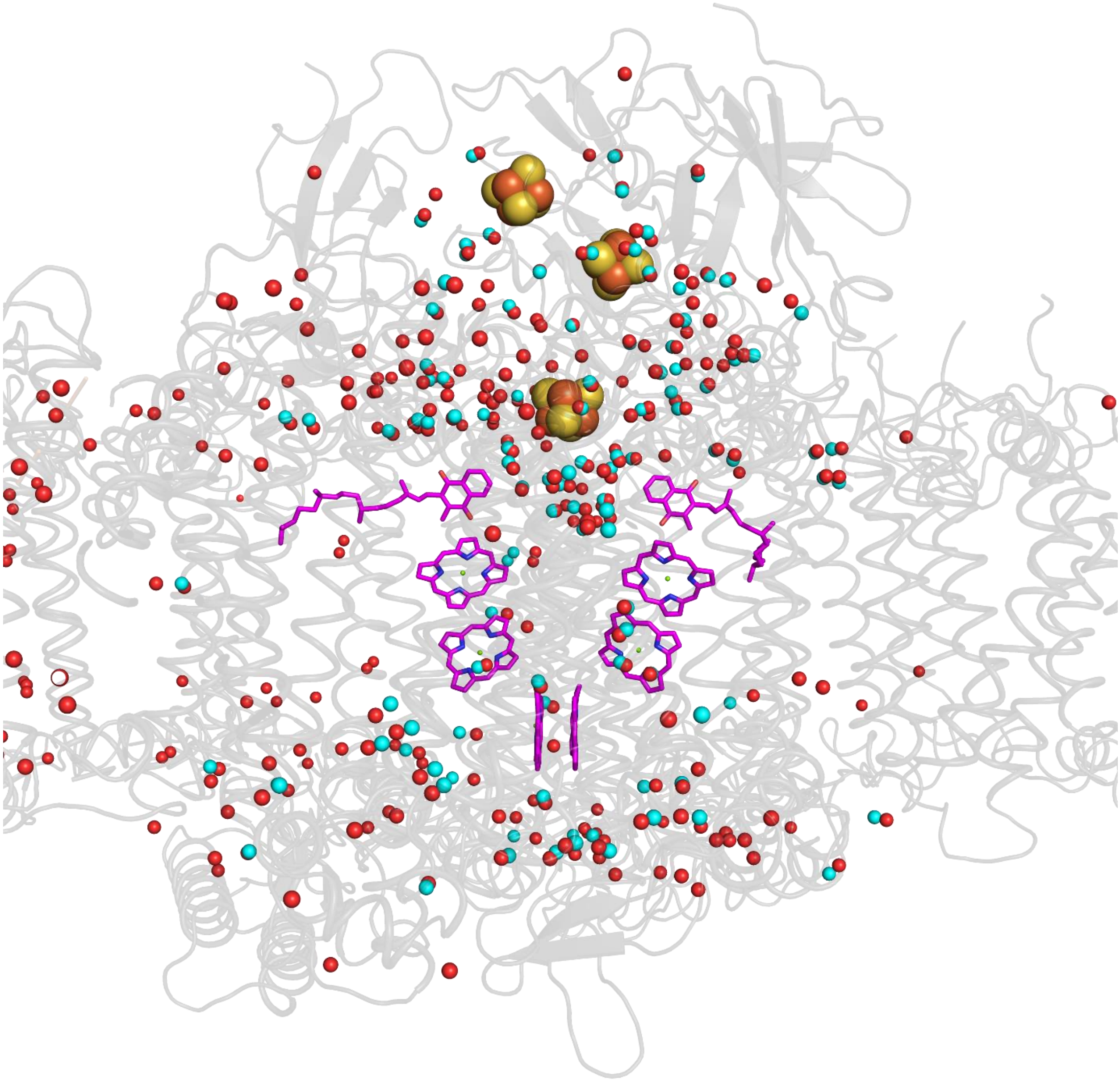

Supplementary Figure 12. The common water of TSP4 and *Synechocystis 6803* PSI. TSP4-10LHC water molecules are shown as red spheres and *Synechocystis* PSI[28] (PDBID 5OY0) are shown as cyan spheres. PSI polypeptides and ETC components are coloured as in Supplementary Figure 8.

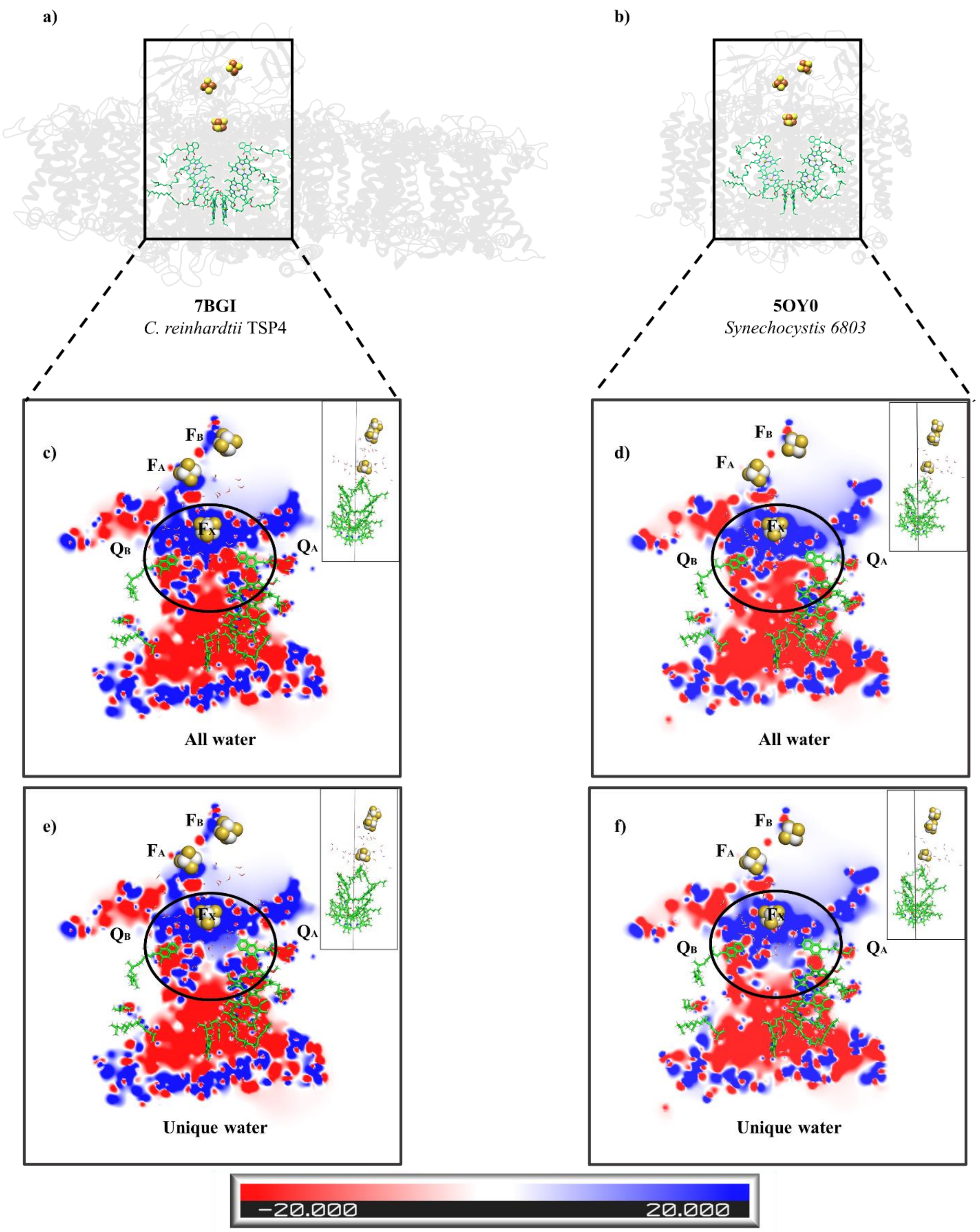

Supplementary Figure 13. The electrostatic potentials of the *C. reinhardtii* TSP4 and *Synechocystis* PSI RC central slice. The structure of *C. reinhardtii* TSP4 (a) and *Synechocystis* PSI (b). For clarity, only the ligands of the proteins (chlorophylls, quinones, and the iron-sulphur clusters) and the water molecules

are presented and are marked with a rectangle. The electrostatic potentials were calculated on two conditions: with all of the water molecules found within a 10 Å radius of FX of *C. reinhardtii* TSP4 (c), or *Synechocystis* (d), or the water molecules that constitute a unique subset to *C. reinhardtii* TSP4 (e), or *Synechocystis* (f). The negative potentials (0 k_BT/e > Φ > -20 k_BT/e) are coloured red, the positive potentials (0 k_BT/e < Φ < 20 k_BT/e) are blue, and the neutral potentials are white (see the colour code at the bottom). The slide location is shown in the small images of the panels, c-through-f. Noticeable changes in the electrostatic potential appear in the region between the quinones and FX, marked by black circles.

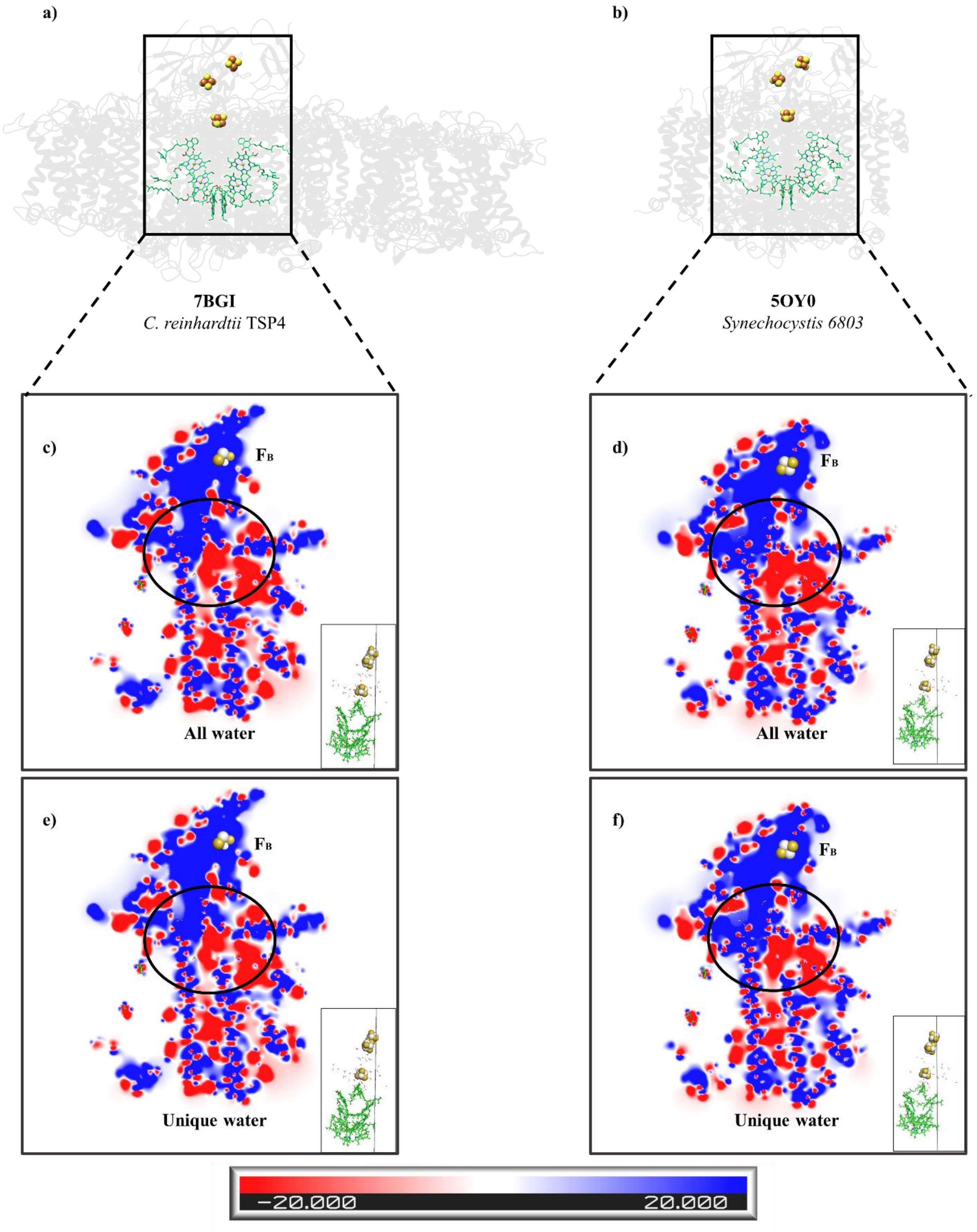

Supplementary Figure 14. The electrostatic potentials of the *C. reinhardtii* TSP4 and *Synechocystis* PSI RC forward slice. The structure of *C. reinhardtii* TSP4 (a) and *Synechocystis* PSI (b). For clarity, only the ligands of the proteins (chlorophylls, quinones, and the iron-sulphur clusters) and the water

molecules are presented and are marked with a rectangle. The electrostatic potentials were calculated on two conditions: with all of the water molecules found within a 10 Å radius of FX of *C. reinhardtii* TSP4 (c), or *Synechocystis* (d), or the water molecules that constitute a unique subset to *C. reinhardtii* TSP4 (e), or *Synechocystis* (f). The negative potentials ($0\ k_BT/e > \Phi > -20\ k_BT/e$) are coloured red, the positive potentials ($0\ k_BT/e < \Phi < 20\ k_BT/e$) are blue, and the neutral potentials are white (see the colour code at the bottom). The slide location is shown in the small images of the panels, c-through-f. The electrostatic potential is similar in this slice, which is nearly devoid of water molecules, marked by black circles.

7BGI
C. reinhardtii TSP4

5OY0
Synechocystis 6803

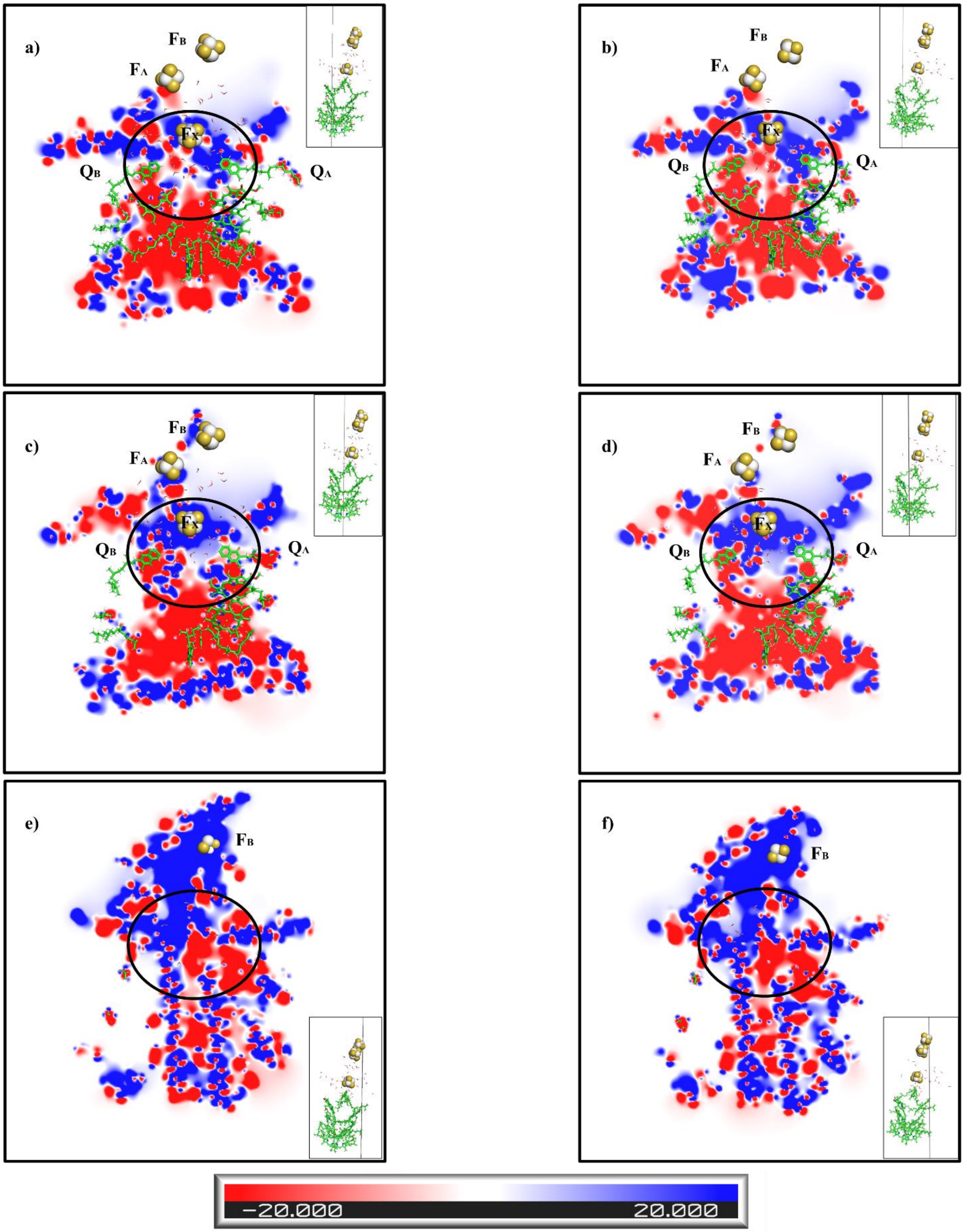

173

Supplementary Figure 15. The electrostatic potentials of the *C. reinhardtii* TSP4 and *Synechocystis* PSI RC, excluding all of the water molecules. The backward slice of *C. reinhardtii* TSP4 (a) and *Synechocystis* PSI (b), the middle slice of *C. reinhardtii* TSP4 (c), or *Synechocystis* (d), or the forward slice of *C. reinhardtii* TSP4 (e), or *Synechocystis* (f.). The negative potentials (0 k_BT/e > Φ > -20 k_BT/e) are coloured red, the positive potentials (0 k_BT/e < Φ < 20 k_BT/e) are blue, and the neutral potentials are white (see the colour code at the bottom). The slide location is shown in the small images of the panels, a-through-f. Removing all of the water molecules results in a similar electrostatic potential in all three views, marked by black circles, compared to the calculated potential shown in Fig.4 and Supplementary Figure 13-14.

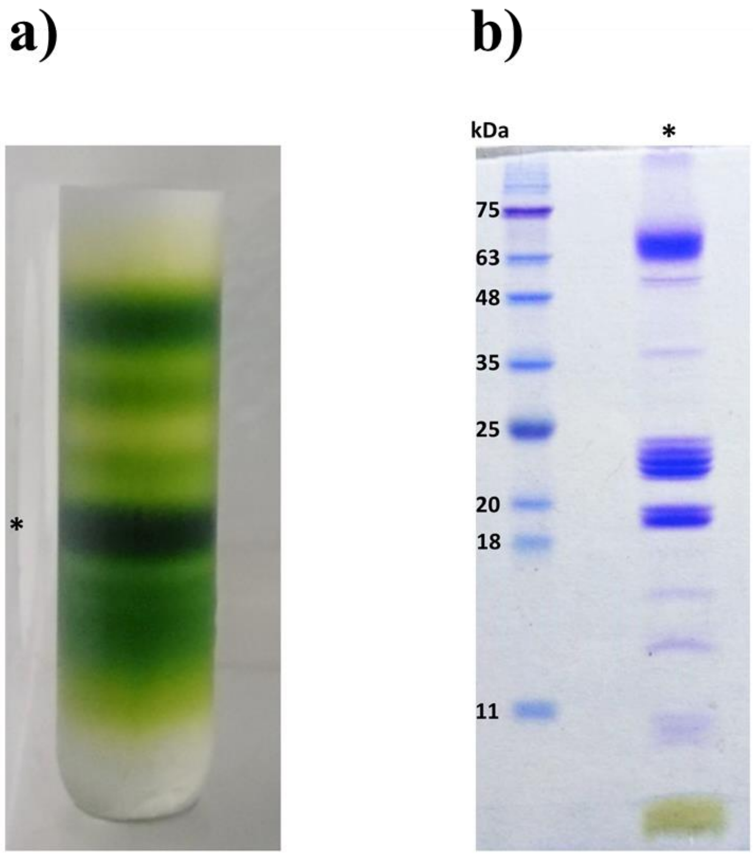

Supplementary Figure 16. TSP4-PSI purification. a) TSP4 sucrose gradient, the retained band is marked with an asterisk. b) SDS-PAGE analysis of the sample that was used for the cryo-EM preparation.

Supplementary Table. 1. Water coordination of TSP4 chlorophylls and their respective H_2O-H_2O-Magnesium angles.

Chain	Number	H_2O-H_2O-MG angle (º)	Chlorophyll type
PsaA	1119	99	a
PsaA	1124	103	a
PsaA	1140	103	a
PsaB	1216	101	a
PsaB	1221	102	a
PsaB	1222	113	a
PsaB	1232	126	a
PsaB	1237	99	a
PsaB	1238	98	a
PsaF	1302	113	a
PsaL	1503	127	a
Lhca1	606	120	a
Lhca1	611	119	a
Lhca1	613	103	a
LhcaZ	610	110	b
LhcaZ	611	106	a
Lhca3	606	129	a
Lhca3	611	105	b
Lhca3	613	92	a
Lhca7	606	130	a
Lhca7	610	101	b
Lhca7	611	114	a
Lhca7	613	106	a
Lhca8	610	99	b
Lhca8	611	116	a
Lhca8	613	91	b
Lhca4	611	110	b
Lhca5	606	141	a
Lhca5	613	92	a
Lhca5	622	106	a
Lhca6	603	106	a
Lhca6	610	103	b
Lhca6	611	100	b
