# Supplementary material for: Dimeric and high-resolution structures of Chlamydomonas Photosystem I from a temperature-sensitive Photosystem II mutant
Source: Commun Biol. 2021 Dec 9;4:1380. doi: 10.1038/s42003-021-02911-7 (PMC8660910; doi:10.1038/s42003-021-02911-7)
Supplement: Supplementary file 2 — Description of Additional Supplementary Files [file 42003_2021_2911_MOESM2_ESM.pdf]

Description of Additional Supplementary Files

File name: Supplementary Movie 1
Description: Electrostatic potentials of the C. reinhardtii TSP4 PSI RC backward slice. The video shows the slice presented in Fig 4, and the electrostatic potential was calculated with all water, unique water, and no water. Slice plane is shown in the small image on the right. ETC chlorophylls and quinones are presented as red sticks, iron-sulphur cluster as brown-white spheres and water molecules as red spheres.

File name: Supplementary Movie 2
Description: Electrostatic potentials of the Synechocystis PSI RC backward slice. The video shows the slice presented in Fig 4, and the electrostatic potential was calculated with all water, unique water, and no water. Slice plane is shown in the small image on the right. ETC chlorophylls and quinones are presented as red sticks, iron-sulphur cluster as brownwhite spheres and water molecules as red spheres.

File name: Supplementary Movie 3
Description: Electrostatic potentials of the C. reinhardtii TSP4 PSI RC central slice. The video shows the slice presented in Extended Data Fig 11, and the electrostatic potential was calculated with all water, unique water, and no water. Slice plane is shown in the small image on the right. ETC chlorophylls and quinones are presented as red sticks, iron-sulphur cluster as brown-white spheres and water molecules Page 7 of 15 as red spheres.

File name: Supplementary Movie 4
Description: Electrostatic potentials of the Synechocystis PSI RC central slice. The video shows the slice presented in Extended Data Fig 11, and the electrostatic potential was calculated with all water, unique water, and no water. Slice plane is shown in the small image on the right. ETC chlorophylls and quinones are presented as red sticks, iron-sulphur cluster as brown-white spheres and water molecules as red spheres.

File name: Supplementary Movie 5
Description: Electrostatic potentials of the C. reinhardtii TSP4 PSI RC forward slice. The video shows the slice presented in Extended Data Fig 12, and the electrostatic potential was calculated with all water, unique water, and no water. Slice plane is shown in the small image on the right. ETC chlorophylls and quinones are presented as red sticks, iron-sulphur cluster as brown-white spheres and water molecules as red spheres.

File name: Supplementary Movie 6
Description: Electrostatic potentials of the Synechocystis PSI RC forward slice. The video shows the slice presented in Extended Data Fig 12, and Page 8 of 15 the electrostatic potential was calculated with all water, unique water, and no water. Slice plane is shown in the small image on the right. ETC chlorophylls and quinones are presented as red sticks, iron-sulphur cluster as brown-white spheres and water molecules as red spheres.

File name: Supplementary Movie 7
Description: Electrostatic potentials of the C. reinhardtii TSP4 and Synechocystis PSI RC backward slice. The video shows the slice presented in Fig 4c-d, and the electrostatic potential was calculated with all water molecules. Slice plane is shown in the small image on the right. ETC chlorophylls and

quinones are presented as red sticks, iron-sulphur cluster as brown-white spheres and water molecules as red spheres.
